# MicroRNA Profile Predicts Recurrence after Resection in Patients with Hepatocellular Carcinoma within the Milan Criteria

**DOI:** 10.1371/journal.pone.0016435

**Published:** 2011-01-27

**Authors:** Fumiaki Sato, Etsuro Hatano, Koji Kitamura, Akira Myomoto, Takeshi Fujiwara, Satoko Takizawa, Soken Tsuchiya, Gozoh Tsujimoto, Shinji Uemoto, Kazuharu Shimizu

**Affiliations:** 1 Department of Nanobio Drug Discovery, Graduate School of Pharmaceutical Sciences, Kyoto University, Kyoto, Japan; 2 Department of Surgery, Graduate School of Medicine, Kyoto University, Kyoto, Japan; 3 New Frontiers Research Laboratories, Toray Industries, Inc. Kanagawa, Japan; 4 Department of Pharmacogenomics, Graduate School of Pharmaceutical Sciences, Kyoto University, Kyoto, Japan; Emory University, United States of America

## Abstract

**Objective:**

Hepatocellular carcinoma (HCC) is difficult to manage due to the high frequency of post-surgical recurrence. Early detection of the HCC recurrence after liver resection is important in making further therapeutic options, such as salvage liver transplantation. In this study, we utilized microRNA expression profiling to assess the risk of HCC recurrence after liver resection.

**Methods:**

We examined microRNA expression profiling in paired tumor and non-tumor liver tissues from 73 HCC patients who satisfied the Milan Criteria. We constructed prediction models of recurrence-free survival using the Cox proportional hazard model and principal component analysis. The prediction efficiency was assessed by the leave-one-out cross-validation method, and the time-averaged area under the ROC curve (ta-AUROC).

**Results:**

The univariate Cox analysis identified 13 and 56 recurrence-related microRNAs in the tumor and non-tumor tissues, such as miR-96. The number of recurrence-related microRNAs was significantly larger in the non-tumor-derived microRNAs (N-miRs) than in the tumor-derived microRNAs (T-miRs, P<0.0001). The best ta-AUROC using the whole dataset, T-miRs, N-miRs, and clinicopathological dataset were 0.8281, 0.7530, 0.7152, and 0.6835, respectively. The recurrence-free survival curve of the low-risk group stratified by the best model was significantly better than that of the high-risk group (Log-rank: P = 0.00029). The T-miRs tend to predict early recurrence better than late recurrence, whereas N-miRs tend to predict late recurrence better (P<0.0001). This finding supports the concept of early recurrence by the dissemination of primary tumor cells and multicentric late recurrence by the ‘field effect’.

**Conclusion:**

microRNA profiling can predict HCC recurrence in Milan criteria cases.

## Introduction

Hepatocellular carcinoma (HCC) is one of the most common malignancies worldwide, and is the fourth most common cause of mortality [1], [2]. In addition, its incidence is increasing in many countries [3], [4]. HCC is difficult to manage, as compared with other common malignant diseases due to the high percentage of co-existing liver cirrhosis. The impaired liver function caused by liver cirrhosis often restricts treatment options, even for early HCC. Liver resection (LR) and orthotopic liver transplantation (OLT) are considered as the only two potentially curative treatment options for HCC. Currently, the Milan Criteria (i.e., solitary tumor ≤5 cm, or 2 or 3 tumors ≤3 cm) [5] are widely accepted as the selection Criteria for OLT in HCC patients. For HCC patients with severe liver cirrhosis (Child-Pugh C), OLT is considered the first-line treatment. In these cases, LR is contraindicated because of impaired liver function. In contrast, there has been an intense debate about which treatment option of LR or OLT is the optimal initial treatment for HCC patients with no to mild liver cirrhosis (Child-Pugh A/B). Some authors have recommended OLT as the first-line treatment for HCC fulfilling the Milan Criteria given the lower tumor recurrence rate after OLT[6], [7]. On the other hand, due to the shortage of donor organs, many liver transplant centers choose LR for HCC patients with Child-Pugh A/B, and who satisfy the Milan Criteria. Therefore, the strategy of a primary LR and salvage transplantation for intrahepatic HCC recurrence is a reasonable tactic for early resectable HCC with preserved liver function. In this strategy, it is important to predict recurrent tumors for selecting the follow-up protocol of patients after LR.

In our previous reports, we screened prognostic factors from various clinical features, and found that co-existing cirrhosis correlated with the outcomes of HCC cases within the Milan Criteria[8]. Moreover, utilizing clinical factors, we also determined a safe expanded selection criteria for the indications of living donor liver transplantation in HCC patients beyond the Milan Criteria[9]. To obtain better-optimized stratification criteria and a more accurate prediction of recurrence, biological information and biomarkers derived from “OMICS” approaches, e.g., transcriptomics and proteomics, will be powerful tools.

MicroRNAs are a class of small non-coding RNAs [19–23 nucleotides (nt)] that have been found in animal and plant cells. As of today, 1049 human microRNAs are registered in the miRBase database (Release 16, September, 2010)[10]–[13]. MicroRNA genes are transcribed as non-coding transcripts, and are processed through a series of sequential steps involving the RNase III enzymes, Drosha and Dicer. The processed microRNAs are finally incorporated into the RNA-induced silencing complex (RISC) to mediate the target mRNA repression of translation and/or degradation. It has been reported that microRNAs are involved in physiological and pathological functions, such as the regulation of developmental timing and pattern formation[14], the restriction of differentiation potential[15], chromatin rearrangements[16], and carcinogenesis[17]. Many of the mechanistic details still remain unknown.

In the last decade, gene expression profiling has been utilized to classify the type of HCC and to predict the recurrence and survival of HCC patients [18]–[21]. Moreover, recent microarray technology has been utilized to analyze a comprehensive microRNA expression profiling of HCC [22], [23]. However, a microarray-based prediction of HCC recurrence, especially for early HCC patients, have not been reported.

Previously, we developed a highly sensitive platforms for both mRNA and microRNA expression profiling [24]. Our ultimate goal is to apply microarray technology into the clinical field. Thus, we previously evaluated a microRNA-microarray platform as a future IVDMIA (*in vitro* diagnostic multivariate index assay) device using analytical procedures recommended by the Microarray quality control (MAQC) project [25]. In this study, we examined whether microRNA microarray technology can predict recurrent HCC after LR for patients who satisfying the Milan Criteria.

## Materials and Methods

### Patients

Between January, 1997 and March, 2007, 639 patients with HCC underwent hepatic resection as a primary curative treatment. Among these patients, 73 patients satisfied the following patient enrollment criteria; 1) no preoperative therapy, 2) within the Milan Criteria, 3) with Child-Pugh A-B level cirrhosis, 4) no or minimum vessel invasion (pathological Vp0-2 and Vv0-1 according to the General Rules for the Clinical and Pathological Study of Primary Liver Cancer, the 4th edition [26]), 5) availability of frozen paired tumor and non-tumor liver tissues, and 6) the quality of extracted RNA was good enough for microarray analysis. This study was approved by the Institutional Review Board of School of Medicine and Kyoto University Hospital, and the Human Tissue Samples Ethics Committee for R&D, Toray Ind., Inc. All patients gave their written informed consent to the sample collection and analyses described in the present study, in agreement with the Declaration of Helsinki.

### RNA extraction

Tumor and non-tumor tissues were obtained and frozen in liquid nitrogen immediately after hepatic resection, and were stored in liquid nitrogen until RNA extraction. The total RNA samples were extracted by a phenol-chloroform RNA extraction method (Trizol; Invitrogen, Carlsbad, Calif., USA). The quality of the purified total RNAs was analyzed by a Bioanalyzer 2100 (Agilent Technologies, Palo Alto, Calif., USA). The criteria for the use of a sample was that the 18s and 28s ribosomal RNA peaks were twice or more higher than the other peaks, as previously described [27]. The RNA Integrity Numbers (RIN) of Bioanalyzer software for all samples was more than 5.

### MicroRNA expression profiling

We utilized Toray's 3D-Gene™ human microRNA chips (miRBase version 12) for microRNA expression profiling. The reproducibility and comparability to Taqman RT-PCR, and the experimental procedures of Toray's microarray, were described previously [25]. Briefly, for each patient, 500 ng of total RNA derived from both tumor and non-tumor samples were labeled using miRCURY LNA™ microRNA Power Labeling Kits Hy5 (Exiqon, Vedbaek, Denmark). The labeled samples were individually hybridized onto the DNA chip surface, and were incubated at 42°C for 16 hours. The washed and dried DNA chip in an ozone-free environment was scanned using a ProScanArray™ microarray scanner (PerkinElmer Inc. Waltham, MA). The obtained microarray images were analyzed using Genepix Pro™ 4.0 software (Molecular Device, Sunnyvale, CA). In this study, the median values of the foreground signal minus the local background were represented as the feature intensities. The microRNA expression profile of all samples was illustrated as a heatmap in Figure S1.

### Data analyses procedures

**Figure 1:**
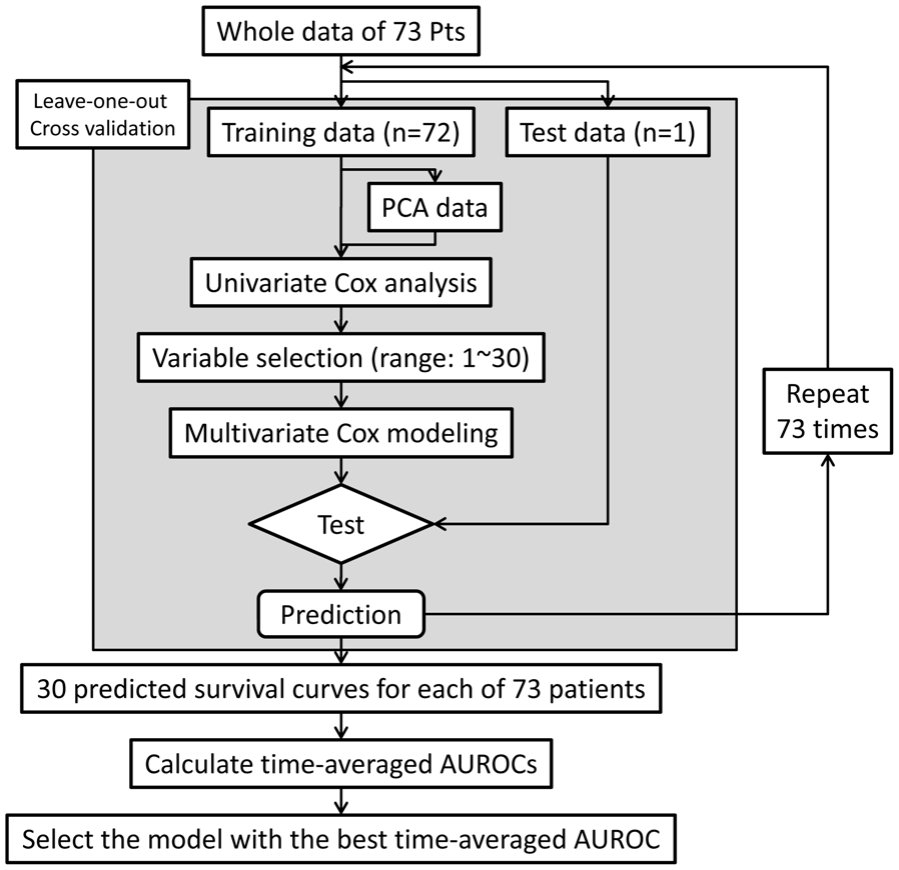
Flow chart of the prediction model construction and validation. To establish prediction models, the leave-one-out cross-validation method (LOOCV), principal component analysis (PCA), and Cox proportional hazard (CoxPH) models were used. In each LOOCV cycle, the variables of the training datasets or principal component (PC) dataset were prioritized by univariate CoxPH model analysis. Next, multivariate CoxPH models were developed using 1∼30 top-ranked variables, and 30 predicted recurrence-free survival curves were the output for a test dataset. After 73 LOOCV cycles, 73×30 predicted recurrence-free survival curves were generated. For each number of variables used, the time-averaged AUROC was calculated using a set of 73 predicted survival curves. Finally, a model with the best time-averaged AUROC was selected.

In this study, the recurrence-free survival was defined as the time between the operation date and the date when the recurrent tumor/tumors were identified. The clinical dataset consists of 63 clinicopathological information points (Table S1). All data obtained from the microarray experiments were normalized by a quantile normalization method [28], and then were filtered (75 percentile of miR expression >6 in log2 scale). Thus, 193 microRNAs were selected, and finally 579 data points (579 = 193×3, T-miR, N-miR, and T/N ratio) for each patient were used for further prediction model construction. The model construction procedure is illustrated in a flow chart (Figure 1). All statistical analyses and prediction model construction were performed using Matlab 2010a software (Mathworks, Natick, MA, USA). The detailed data process procedures are described in a supplemental file. In this study, p-values less than 0.05 were considered as statistically significant. All microRNA microarray data were registered into NCBI's Gene Expression Omnibus (GEO) database (http://www.ncbi.nlm.nih.gov/projects/geo/). The accession numbers are GSE21362 for series IDs, and GSM533698∼533843 for sample IDs.

## Results

### Patients' characteristics

Table 1 summarizes the clinicopathological characteristics of the patients. All patients were treated with curative surgical liver resection. The mean recurrence-free survival of the complete cases (n = 45) was 618 days (standard deviation: 517 days). The mean follow-up periods of the censored cases (n = 28) was 1902 days. Among the 73 patients, 45 had recurrent tumors. The types of initial recurrences were intrahepatic, extrahepatic, and both for 41, 2, and 2 patients, respectively. According to univariate Cox proportional hazard model (CoxPH) analysis, T-factor and HBV infection are significantly associated with a shorter recurrent-free survival (*P* = 0.0131 and 0.0490, respectively).

**Table 1:** Clinical Features of 73 HCC patients within Milan Criteria and Univariate Cox Proportional Hazard Model.

			Cox proportional hazard model	
Clinical features			hazard ratio	p-value
Age	mean, range			
		65.8 (40–88)	0.981	0.228
Gender				
	female	19	1.077	0.825
	male	54		
Tumor size (Max diameter)	mean±SD			
		3.2±1.0	0.833	0.235
Child-Pugh classification				
	A	69	1.924	0.277
	B	4		
Cirrhosis				
	(-)	36	1.246	0.463
	(+)	37		
Tumor Grade				
	well	21	0.982	0.938
	moderate	45		
	poor	7		
T factor				
	T1	7	1.692	0.0131
	T2	51		
	T3	13		
	T4	2		
HBV antigen				
	(-)	61	2.033	0.0490
	(+)	12		
HCV antibody				
	(-)	22	1.119	0.734
	(+)	51		

Tumor Grade and Tumor factor are recorded according to the General Rules for the Clinical and Pathological Study of Primary Liver Cancer (26).

### Recurrence-related microRNA signature

**Figure 2:**
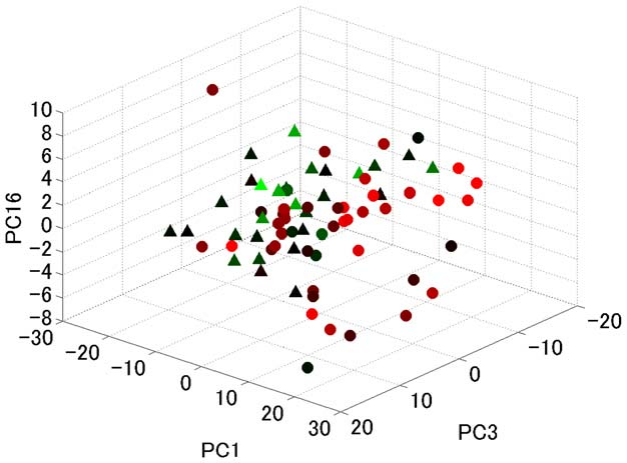
Three-dimensional scatter plot of PCA. The x-, y-, and z-axes represent the top-3 ranked PCs (PC3, PC16 and PC1). The color graduation scale from green, black to red represent short, intermediate, and long recurrence-free survival, respectively. The closed circles, and closed triangles correspond to complete and censored cases, respectively. Patients with poor outcomes tend to be in the right-lower side from this point of view, whereas patients with good outcomes tend to be in left-upper side.

As the first step in this analysis, we utilized univariate CoxPH to identify recurrence-related microRNAs (Table 2). Interestingly, most of the identified tumor-derived microRNAs (T-miRs) were negatively associated with HCC recurrence, which suggests that tumor-suppressor microRNAs are down-regulated in tumor tissues. On the other hand, most of the identified non-tumor-derived microRNAs (N-miRs), which are ranked in the top-20, were positively associated with HCC recurrence, which indicated that oncogenic microRNAs are predominantly up-regulated in non-tumor tissues. In addition, the number of miRs significantly correlated with HCC recurrence was larger in non-tumor tissues than in tumor tissues (Fisher's exact test: p<0.0001). As the second step, we applied principal component analysis (PCA) to the univariate CoxPH analysis. The five principal components (PCs) of the microRNA expression profile were significantly correlated to the recurrence-free survival of HCC (Table 3). To show the clinical significance of each PC, the correlated clinical features of each PC are also listed in Table 3. For example, PC3 is a HBV and AFP related PC, and PC16 is a T-factor related one. A 3-dimentional scatter plot shows that the top 3 ranked PCs correlated to the recurrence-free survival of HCC patients (Figure 2).

**Table 2:** Recurrence-related microRNAs.

	Recurrence-related microRNAs in tumor tissues						Recurrence-related microRNAs in non-tumor tissues (Top 20 out of 56 significant miRs)				
Rank	microRNA	hazard ratio	miR-type	p-value	FDR	Rank	microRNA	hazard ratio	miR-type	p-value	FDR
1	miR-100	0.5170	Ts-miR	<0.0001	0.011	1	miR-27a	7.4696	OncomiR	<0.0001	0.004
2	miR-99a	0.6318	Ts-miR	0.0006	0.035	2	miR-24	11.5691	OncomiR	0.0001	0.004
3	miR-99b	0.6087	Ts-miR	0.0013	0.040	3	miR-96	1.4868	OncomiR	0.0002	0.003
4	miR-125b	0.8062	Ts-miR	0.0028	0.048	4	miR-21	1.6847	OncomiR	0.0004	0.006
5	miR-378	0.6339	Ts-miR	0.0043	0.055	5	miR-18a	1.9046	OncomiR	0.0007	0.007
6	miR-129-5p	0.6888	Ts-miR	0.0075	0.083	6	miR-23a	5.5879	OncomiR	0.0010	0.007
7	miR-125a-5p	0.7441	Ts-miR	0.0089	0.080	7	miR-18b	1.6114	OncomiR	0.0013	0.007
8	miR-497	0.7737	Ts-miR	0.0123	0.090	8	miR-142-3p	1.4802	OncomiR	0.0018	0.007
9	miR-22	0.5470	Ts-miR	0.0141	0.085	9	miR-362-3p	1.4718	OncomiR	0.0020	0.006
10	miR-140-3p	0.5603	Ts-miR	0.0306	0.208	10	miR-1202	0.7592	Ts-miR	0.0021	0.006
11	miR-145	0.7531	Ts-miR	0.0341	0.213	11	let-7e	3.3158	OncomiR	0.0025	0.004
12	miR-221	1.4608	OncomiR	0.0441	0.266	12	let-7f	5.6499	OncomiR	0.0039	0.009
13	miR-195	0.7803	Ts-miR	0.0487	0.262	13	miR-191	7.4922	OncomiR	0.0047	0.011
						14	miR-107	6.0708	OncomiR	0.0057	0.014
						15	miR-148a	0.5922	Ts-miR	0.0061	0.013
						16	miR-222	1.5374	OncomiR	0.0077	0.013
						17	miR-103	5.5941	OncomiR	0.0086	0.012
						18	miR-126*	2.2102	OncomiR	0.0087	0.012
						19	miR-425	2.1610	OncomiR	0.0089	0.012
						20	miR-378	0.6406	Ts-miR	0.0090	0.011

This table lists hazard ratio and p-value calculated by an univariate Cox's propotional hazard model for each microRNA. MicroRNAs which hazard ratio is greater than 1 were correlated with frequent recurrence, and are potential **oncomiRs**. In contrast, microRNAs with hazard ratio less than 1 were associated with good recurrence-free survivals, and would be a tumor-suppressor miRs (**Ts-miR**). The number ( = 56) of recurrence-related microRNAs in non-tumor tissues is significantly larger than that ( = 13) in tumor tissues (Fisher's exact t-test: p<0.00001). False discovery rate (FDR) were estimated by permutation analysis.

**Table 3:** Recurrence-related principal components and correlated clinical factors.

		Cox proportional hazard model			
Rank	Principal components	hazard ratio	p-value	Notable correlated clinical factors	p-value
1	PC3	0.9592	0.0171	serum, AFP*	<0.0001
				serum, total-bilirubin*	0.0009
				HBV antigen§	0.0020
2	PC16	0.9044	0.0207	platelet*	0.0040
				Max diameter of tumor*	0.0135
				T-factor (clinical)**	0.0202
3	PC1	1.0335	0.0244	serum, PIVKA-II*	0.0018
				gender§	0.0398
4	PC4	1.0514	0.0246	HCV antibody§	0.0002
				serum, AST*	0.0037
				platelet*	0.0071
5	PC5	0.9591	0.0434	capsule formation (pathological)§	<0.0001
				presence of liver cirrhosis§	0.0042
				ICG-R15*	0.0198

*: continuous variables,

**: category variables,

§: dichotonized variables.

The p-values of correlation between PCs and clinical factors were calculated by Pearson's correlation analysis, Kruskal-Wallis test, and t-test for continuous, category, and dichotonized variables, respectively.

### Constructing prediction models of HCC recurrence-free survival

**Figure 3:**
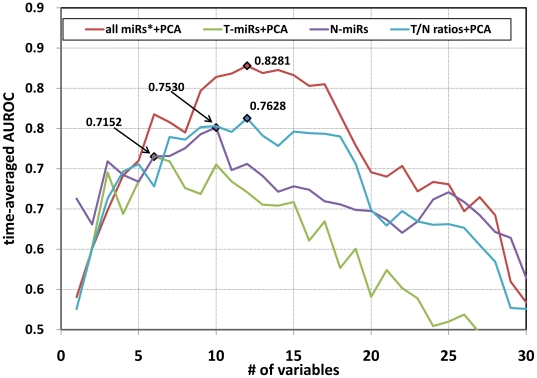
Changes in time-averaged AUROCs and the number of variables used for prediction model construction. The prediction models of HCC recurrence were constructed by the LOOCV method. The prediction accuracy for each model was evaluated by time-averaged AUROC between 6 months and 5 years after the operation. The 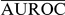 were different depending upon the sample tissues, the inclusion or exclusion of PCA, and the number of variables used for constructing the prediction models. The best prediction accuracy was achieved when the model was construct using top-twelve PCs data derived from all microRNA data (*: expression data of T-miRs and N-miRs, and T/N ratios). The best models using T-miRs, N-miRs, and T/N ratios were obtained from the top-6 PCs, top-10 microRNAs, and top-12 PCs, respectively.

**Figure 4:**
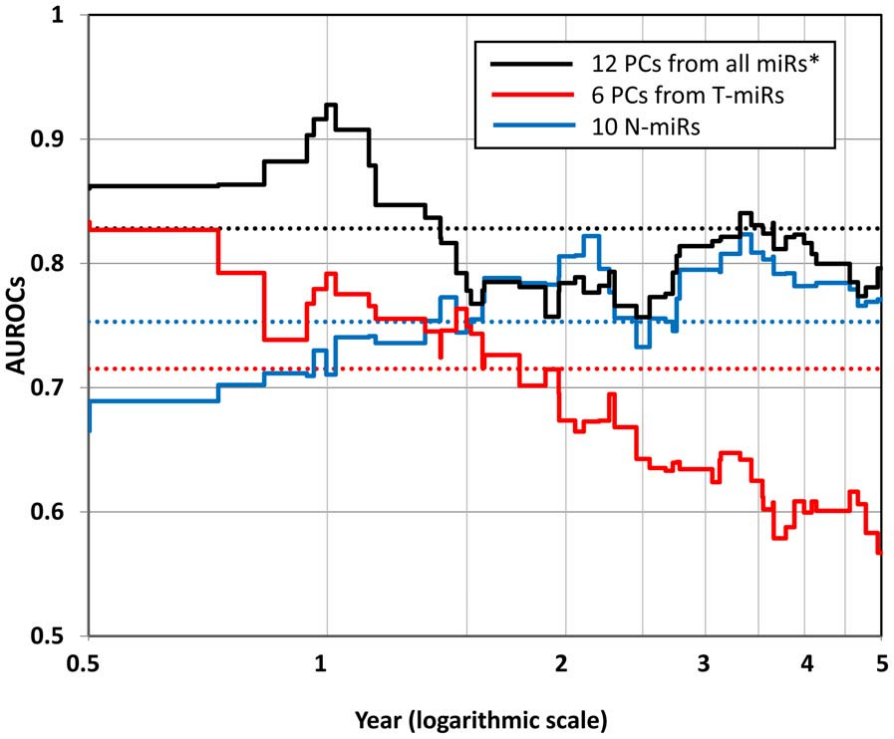
The AUROC changes of the best prediction model between 6 months and 5 years. In general, the prediction accuracy depends upon the time point used to evaluate the prediction accuracy of the survival curve. Thus, we adopted 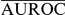 as an index of prediction accuracy for the survival curve. To cover both early and late recurrences, we calculated the 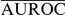 between 6 months and 5 years. The black step graph shows the changes in the AUROCs for the overall best prediction model (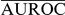 = 0.8281). The red and blue step graphs represent the changes of AUROCs of the best models using T-miRs and N-miRs datasets. The dotted lines of each color correspond to the 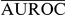 for each step graph. This prediction model using T-miRs can predict early recurrences better, whereas the model using N-miRs is better for late recurrences.

**Figure 5:**
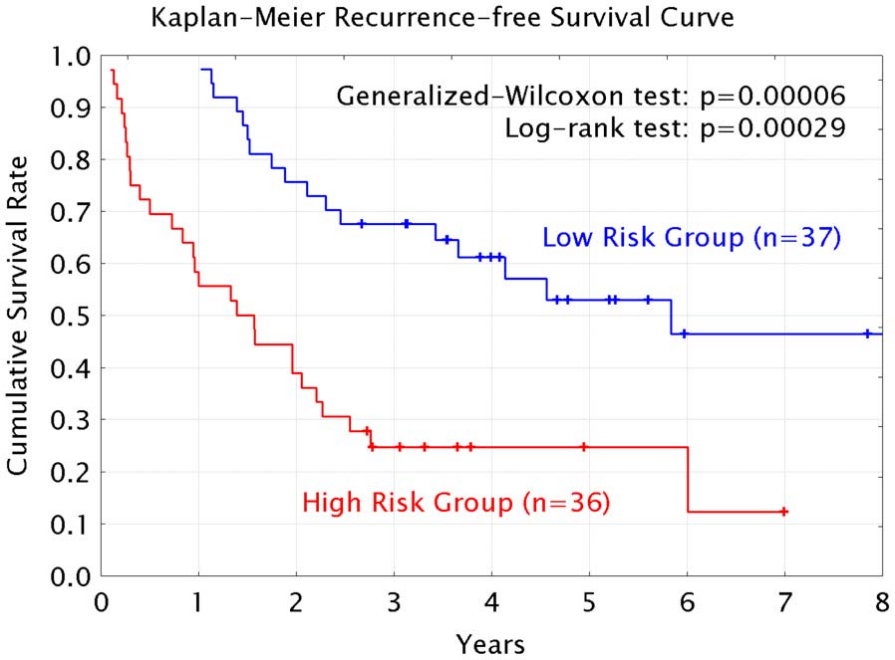
Kaplan-Meier cumulative recurrence-free survival curves. According to the predicted recurrence-free survival rate, the patients were stratified as low (n = 37) and high (n = 36) risk groups. The recurrence-free survival of the low risk group was significantly better than that of high risk group by both a generalized-Wilcoxon test and the log-rank test.

The prediction model was constructed by multivariate CoxPH models with leave-one-out cross-validation method (Figure 1). The accuracy of each prediction was evaluated by a time-averaged AUROC (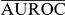) between 6 months and 5 years. The 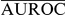 changes depending upon the sample tissues, inclusion or exclusion of PCA, the number of variables used for constructing prediction models. Among the various conditions, the best 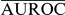 ( = 0.8281) was achieved when the model was constructed using top-12 PCs data generated from an integrated dataset consisting of T-miRs and N-miRs, and T/N ratios (Figure 3). Figure 4 shows the change of AUROCs for different time points between 6 months and 5 years after LR. This model can predict early recurrences better (the best AUROC = 0.9276 at 1 year after LR) than late recurrences. Next, according to this prediction model, the 73 cases were stratified into Low- (n = 37) and High-risk (n = 36) groups. Consequently, the Kaplan-Meier recurrence-free survival curve of the Low-risk group was significantly better than the High-risk group (Figure 5, Wilcoxon test: p = 0.00006, log-rank test: p = 0.00029).

The best prediction models generated from only the clinical data (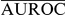 = 0.6835), T-miRs data (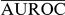 = 0.7152), and N-miRs data (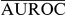 = 0.7530) were obtained when 2 PCs, 6 PCs, and 10 individual microRNAs was used. The overall best prediction model predicts recurrences significantly better than these three models (paired t-test: p<0.00001). In addition, the 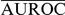 of the N-miRs dataset was also significantly higher than that of T-miRs dataset. As shown in Figure 4, the AUROCs of the T-miRs models gradually decreased over the time course between 6 months and 5 years (Pearson's p-value <0.0001), which indicates that the T-miRs can predict early recurrences better than late recurrences. In contrast, the AUROCs of the N-miR models gradually elevated, which suggests that N-miRs can predict late recurrences better than early recurrences (Pearson's p-value <0.0001).

### Contribution analysis of microRNAs in the best prediction model

The best overall model was constructed with the PCA and CoxPH model. Since the coefficients of the CoxPH model were for PCs not for individual microRNAs, it is difficult to determine how much each microRNA contributes to this prediction model. In this study, we combine the coefficients of the PCA and CoxPH, and converted them to a hazard ratio formula by PC values into individual microRNAs expression values. As shown in the supplemental Description S1, the coefficient of the i-th microRNA can be expressed as 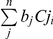, where *n*, *b_j_*, and *Cj_i_* represent the number of PCs, the beta value of the j-th PC, and the coefficient of the i-th microRNA in the j-th PC, respectively. Table 4 shows the top-20 contributory microRNAs or Tumor/Non-tumor ratios (T/N ratios) that are positively and negatively correlated with HCC recurrence. Interestingly, the expressions of miR-96 in non-tumor tissues and miR-96 T/N ratio are the top ranked microRNAs that are positively and negatively associated with HCC recurrence, respectively. To understand how miR-96 plays biological roles regarding HCC recurrence, it is important to identify target genes of miR-96. In this study, we identified three genes, LRP6, FOXO1A, and MAP2K1, that meet several criteria, such as, 1) possessing cancer-related functions according to an ontology database, 2) having microRNA target sites predicted by Target Scan v.5.1 (www.targetscan.org/) in their 3′-UTR, 3) inverse correlation to miR-96 expression level in 146 samples (tumor and non-tumor paired samples derived from 73 patients), (Table S2). Furthermore, we also checked whether the miR-96 expression data by DNA chips was reliable or not. The expression data of miR-96 by Taqman RT-PCR assay was significantly correlated with that by DNA chip analysis (Figure S2).

**Table 4:** Contribution analysis of individual microRNAs in the overall best prediction model.

	microRNAs positively associated with recurrence				microRNAs negatively associated with recurrence			
Rank	miR name	Tissue type	Coefficient	95%CI†	miR name	Tissue type	Coefficient	95%CI†
1	miR-96 §	N	0.1349	(0.1259∼0.1449)	miR-96	T/N ratio	−0.0865	(−0.0941∼−0.0793)
2	miR-139-5p	T/N ratio	0.0750	(0.0661∼0.0850)	miR-374b	T/N ratio	−0.0785	(−0.0861∼−0.0710)
3	miR-139-5p	T	0.0727	(0.0646∼0.0815)	miR-182	T/N ratio	−0.0604	(−0.0659∼−0.0549)
4	miR-126*	T	0.0676	(0.0620∼0.0737)	miR-378 §	N	−0.0561	(−0.0612∼−0.0511)
5	miR-142-3p	T	0.0673	(0.0599∼0.0749)	miR-193b	T/N ratio	−0.0543	(−0.0600∼−0.0493)
6	miR-142-3p	T/N ratio	0.0659	(0.0575∼0.0754)	miR-193b	T	−0.0539	(−0.0596∼−0.0483)
7	miR-362-3p	T	0.0596	(0.0538∼0.0659)	miR-214	T/N ratio	−0.0535	(−0.0585∼−0.0489)
8	miR-374b	N	0.0563	(0.0499∼0.0629)	miR-125b §	T	−0.0522	(−0.0569∼−0.0479)
9	miR-10b	T	0.0548	(0.0482∼0.0616)	miR-125b	T/N ratio	−0.0521	(−0.0565∼−0.0483)
10	miR-200a	N	0.0544	(0.0486∼0.0599)	miR-99b	T/N ratio	−0.0517	(−0.0564∼−0.0474)
11	miR-224	N	0.0530	(0.0466∼0.0599)	miR-1202 §	N	−0.0515	(−0.0584∼−0.0451)
12	miR-483-3p	T	0.0529	(0.0459∼0.0602)	miR-18b	T/N ratio	−0.0513	(−0.0590∼−0.0440)
13	miR-200a	T	0.0527	(0.0477∼0.0580)	miR-365	T/N ratio	−0.0511	(−0.0573∼−0.0454)
14	miR-1202	T/N ratio	0.0507	(0.0441∼0.0574)	miR-100	T/N ratio	−0.0506	(−0.0559∼−0.0456)
15	miR-96	T	0.0484	(0.0423∼0.0543)	miR-365	T	−0.0504	(−0.0563∼−0.0445)
16	miR-665	T/N ratio	0.0474	(0.0413∼0.0534)	miR-210	T	−0.0502	(−0.0567∼−0.0440)
17	miR-1274a	T/N ratio	0.0472	(0.0428∼0.0518)	miR-100 §	T	−0.0500	(−0.0547∼−0.0459)
18	miR-10b	T/N ratio	0.0471	(0.0408∼0.0533)	miR-214	T	−0.0491	(−0.0544∼−0.0443)
19	miR-665	T	0.0469	(0.0411∼0.0528)	miR-378 §	T	−0.0489	(−0.0536∼−0.0444)
20	miR-1228	T/N ratio	0.0446	(0.0398∼0.0500)	miR-182	T	−0.0476	(−0.0534∼−0.0419)

Positive and negative coefficients indicates that higher and reduced expression of microRNA is associated with recurrence, respectively.

§ : overlapped microRNAs in Table 2.

† : CI, confidence interval, calculated by 1000 times bootstrap resampling.

### Differentially expressed microRNAs

Differentially expressed microRNAs between T-miRs and N-miRs are shown in Table S3, S4. Among 73 cases enrolled in this study, 4 patients had macroscopically normal and pathologically normal (n = 1) or slightly fibrotic (n = 3) liver tissues. We listed differentially expressed microRNAs compared to these four normal liver samples in Table S5. According to Table S3, S4, S5, miR-96 expression level was gradually elevated from normal liver, via non-tumor liver tissues with accompanied chronic change, to tumor tissues. We also listed differentially expressed microRNAs depending upon HBV/HCV status and tumor differentiation grade in Table S6, S7, S8. Furthermore, using univariate Cox proportional hazard model, we show microRNAs significantly associated with HCC recurrence in subgroups categorized by HBV/HCV status and tumor differentiation grade in Table S9, S10, S11, S12.

## Discussion

In this study, we performed microRNA signature analysis and developed a mathematical model to predict HCC recurrence in patients within the Milan Criteria and with mild liver cirrhosis (i.e., Child-Pugh A/B). To date, there are many microarray-based studies [18]–[21] that predict the outcomes of HCC patients. However, our study is the first microarray-based study that focuses on a potential population of patients for future salvage transplantation, and that underwent liver resection and fulfill the Milan Criteria.

### Clinical significance of HCC recurrence prediction for patients within the Milan Criteria

HCC patients who satisfy the Milan Criteria and who have sufficient liver function have two potential curative therapeutic options: liver resection and orthotopic liver transplantation. However, both options have advantages and disadvantages. Therefore, a compromised option of liver resection plus salvage liver transplantation seems reasonable. Even taking this option, recurrent tumors must be detected in the early phase; otherwise, the benefits of salvage transplantation would be diminished. Performing intensive follow-ups for all LR patients is not feasible. Therefore, a strategy of more detailed follow-ups for patients with higher risk is necessary. This prediction model will be a helpful tool to stratify these patients.

In this study, we obtained a relatively high prediction accuracy (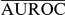  = 0.8281) using a combined dataset of T-miRs and N-miRs. Previously, many prediction models of HCC outcomes have been reported. However, it is very difficult to compare the prediction accuracy to other models, because the target population in the HCC patients from this study was different from those in other reports.

### Significance of microRNA profile in tumor and non-tumor tissues

In this analysis, the prediction efficiency constructed using the N-miRs dataset was better than that constructed using the T-miRs. It is reasonable, because there are more recurrence-related microRNAs in the N-miRs than in the T-miRs, according to univariate CoxPH model analyses (Table 2). Previously, we reported that co-existing cirrhosis was associated with a higher recurrence rate after hepatic resection of HCC within the Milan Criteria. This finding suggests that recurrences due to the dissemination of primary tumors cells occur more often within the initial few years after resection, and that late recurrences in cirrhotic livers are likely attributable to multicentric de novo carcinogenesis rather than dissemination of the primary tumor. In this study, as shown in Figure 4, the T-miR models can predict early recurrences better than late recurrences, whereas the N-miR models can predict late recurrences better than early recurrences. This finding suggests that the T-miR profile would represent the malignant potential of primary tumors, and would be associated with the presence of dissemination and the consequent early recurrence, and that the N-miR profile would reflect the accumulation of genome abnormalities (the ‘field effect’) in the remaining non-cancerous liver cells, and would be associated with multicentric late recurrence. Recently, several papers have reported that an abnormal mRNA expression profile in non-cancerous tissues is associated with HCC recurrence [21], [29], [30]. Moreover, our previous clinical study showed that co-existing cirrhosis correlated with the prognosis and recurrence of HCC within the Milan Criteria [8]. Therefore, our finding derived from the microRNA profile also supports the concept of early and late recurrence that these previous reports have proposed.

### HBV infection, miR-96, and HCC Recurrence

The contribution analysis of microRNAs in the best prediction model demonstrated that miR-96 in non-tumor tissues was the most strongly associated with HCC recurrence. Previously, miR-96 was identified as HBV infection-related microRNA[31]. In this study, HBV infection and HBV-related PC3 were significantly correlated with HCC recurrence, according to the univariate CoxPH model (Table 2–3). Thus, it was reasonable that miR-96 was identified as a recurrence-related microRNA. In a supplement analysis, we identified three genes, LRP6, FOXO1A, and MAP2K1, that meet several criteria, described in result section. Among them, the TargetScan predicted that FOXO1A has the highest context score (an index for strength of bond between mature microRNA and target sites to miR-96. Despite a strong inverse correlation with miR-96 expression, LRP6 and MAP2K1 were predicted to have the relatively lower context scores than FOXO1A. Taken together, FOXO1A would be the most important HCC-related target gene of miR-96 microRNAs. This finding was concordant with a previous report[32].

### Time-averaged AUROCs as an index for survival prediction accuracy

The CoxPH model can analyze data containing censored cases, and can predict the survival curves of test cases using a baseline survival curve by calculating a hazard ratio. However, it is difficult to evaluate the prediction accuracy of CoxPH models. Usually, analysts stratify the patients according to the CoxPH model predictions, and then compare the difference between groups using a generalized-Wilcoxon or log-rank tests, or calculate the sensitivity, specificity, or AUROC at a given time point, such as the 2-year survival. However, the prediction accuracy of survival models depends upon stratification criteria of the patients and the selection of a prediction time point. Therefore, the prediction accuracy of survival models may be under- or over-estimated. Thus, we proposed time-averaged AUROC as a new evaluation index for the prediction efficiency of the CoxPH model, which will be a robust index.

### Future prospects regarding clinical application of microarray technology

The MAQC project was initiated by the U.S. FDA, and the second phase (MAQC-II) is currently in progress [33]. Its aims are to assess the capabilities and limitations of microarray-based predictive models, and to reach a consensus for the development and validation of microarray-based predictive models for personalized medicine. Although reports from MAQC-II have not been published yet, their reports will have a great impact on finalizing a draft guideline of IVDMIA. Prior to finalizing the IVDMIA guideline by the U.S. FDA, the FDA have already cleared two microarray-based IVDMIA devices, MammaPrint™ and Pathwork Tissue Origin Test™. In addition, OncoTypeDX®, a RT-PCR-based IVDMIA, is widely used because health insurance companies have adopted it as a criterion for insurance payment. However, a microRNA-based IVDMIA device has not been developed and approved yet. MicroRNA can be detected even in formalin-fixed paraffin-embedded specimens [34], or remote fluid samples such as blood[35], because of their stability. Therefore, microRNAs are thought to be good biomarkers. The progress in this study will be fundamental for the future application of microRNA-microarray based IVDMIA into the clinical field. However, our prediction model was only internally validated. Therefore, prospective and external validation is necessary before it is introduced to put microRNA microarray into practical use.

## Supporting Information

Description S1 Details of analysis procedures were described.
(DOC)

Figure S1 A heatmap and unsupervised clustergram of microRNA expression in tumor and non-tumor tissues of HCC patients. This heatmap represents an overview of microRNA expression profile. The microRNA expression data was centered by 2 directions (i.e., by genes and patients). Red, green, and black represent high, low, and intermediate microRNA expression. Blue and yellow bars on the top of the heatmap represent non-tumor, and tumor tissues.
(DOC)

Figure S2 Correlation between DNA chip and Taqman data for miR-96 expression. DNA chip expression data for miR-96 were validated by Taqman microRNA assay. DNAchip data and Taqman data were significantly correlated (p<0.0001). Left: x-axis: DNA chip data in a log2 scale, y-axis: Taqman data in an arbitrary log2 scale. Right: x-axis: DNA chip data in a log2 scale, y-axis: Taqman data in a log2 scale.
(DOC)

Table S1 Variables in clinicopathological dataset.
(DOC)

Table S2 Putative miR-96 target genes which expression is inversely correlated with miR-96 expression. *: these values are provided by TargetScan v.5.1, **: rank of total context score among 787 putative miR-96 target genes predicted by TargetScan. †: Pearson's correlation coefficients and p-values, ‡: Pubmed hit count using key words of gene symbol and “HCC, liver cancer, hepatocellular carcinoma”, accessed on Dec 26, 2010.
(DOC)

Table S3 Significantly up-regulated microRNAs in HCC tumor tissues compared to non-tumor tissues. Up-regulated microRNAs with p<0.01 are listed. T-miRs, N-miRs: mean values of each T-miR and NmiR expression in log2 scale, fold change: expression ratio of each T-miR compared with corresponding N-miR, p-value: p-values of paired T-test. Order of microRNA is sorted by fold-change.
(DOC)

Table S4 Significantly down-regulated microRNAs in HCC tumor tissues compared to non-tumor tissues. Down-regulated microRNAs with p<0.01 are listed. T-miRs, N-miRs: mean values of each T-miR and NmiR expression in log2 scale, fold change: expression ratio of each T-miR compared with corresponding N-miR, p-value: p-values of paired T-test. Order of microRNA is sorted by fold-change.
(DOC)

Table S5 Differentially expressed microRNA compared with normal liver tissues. Differentially expressed microRNAs with p<0.05 are listed. T-miRs, N-miRs: mean values of each T-miR and NmiR expression in log2 scale, fold change: expression ratio of each T-miR or N-miR compared with normal liver tissues (n = 4), p-value: p-values of unpaired T-test. The miR order is sorted by fold-change.
(DOC)

Table S6 Differentially expressed microRNAs depending upon HBV status. *: p-values of Student's T-test. Differentially expressed microRNAs with p<0.05 are listed.
(DOC)

Table S7 Differentially expressed microRNAs depending upon HCV status. * p-values of Student's T-test. Differentially expressed microRNAs with p<0.05 are listed.
(DOC)

Table S8 Differentially expressed microRNAs depending upon cellular grade. *: p-values of one-way ANOVA test. Differentially expressed microRNAs with p<0.05 are listed.
(DOC)

Table S9 Recurrence related microRNAs in HBV-positive cases. Univariate Cox proportional hazard model identified microRNAs associated with poor (red) and better (blue) recurrent outcome, respectively. Top-twenty significant microRNAs with p-value <0.05 are listed. MicroRNAs (displayed in red) which hazard ratio is greater than 1 were correlated with frequent recurrence, and are potential oncomiRs. In contrast, microRNAs (shown in blue) with hazard ratio less than 1 were associated with good recurrence-free survivals, and would be a tumor-suppressor miRs.
(DOC)

Table S10 Recurrence related microRNAs in HCV-positive cases. Univariate Cox proportional hazard model identified microRNAs associated with poor (red) and better (blue) recurrent outcome, respectively. Top-twenty significant microRNAs with p-value <0.05 are listed. MicroRNAs (displayed in red) which hazard ratio is greater than 1 were correlated with frequent recurrence, and are potential oncomiRs. In contrast, microRNAs (shown in blue) with hazard ratio less than 1 were associated with good recurrence-free survivals, and would be a tumor-suppressor miRs.
(DOC)

Table S11 Recurrence related microRNAs in hapatitis virus-negative cases. Univariate Cox proportional hazard model identified microRNAs associated with poor (red) and better (blue) recurrent outcome, respectively. Top-twenty significant microRNAs with p-value <0.05 are listed. MicroRNAs (displayed in red) which hazard ratio is greater than 1 were correlated with frequent recurrence, and are potential oncomiRs. In contrast, microRNAs (shown in blue) with hazard ratio less than 1 were associated with good recurrence-free survivals, and would be a tumor-suppressor miRs.
(DOC)

Table S12 Recurrence related microRNAs in grade 1–2 HCC cases. Univariate Cox proportional hazard model identified microRNAs associated with poor (red) and better (blue) recurrent outcome, respectively. Top-twenty significant microRNAs with p-value <0.05 are listed. MicroRNAs (displayed in red) which hazard ratio is greater than 1 were correlated with frequent recurrence, and are potential oncomiRs. In contrast, microRNAs (shown in blue) with hazard ratio less than 1 were associated with good recurrence-free survivals, and would be a tumor-suppressor miRs.
(DOC)
