# Supplementary material for: MicroRNA Profile Predicts Recurrence after Resection in Patients with Hepatocellular Carcinoma within the Milan Criteria
Source: PLoS One. 2011 Jan 27;6(1):e16435. doi: 10.1371/journal.pone.0016435 (PMC3029327; doi:10.1371/journal.pone.0016435)
Supplement: Description S1 — Details of analysis procedures were described. (DOC) [file pone.0016435.s001.doc]

**Supplemental description**

**MicroRNA Profile Predicts Recurrence After Resection in Patients with Hepatocellular Carcinoma within the Milan Criteria.**

Fumiaki Sato^1^, Etsuro Hatano^2^, Koji Kitamura^2^, Akira Myomoto^3,4^, Takeshi Fujiwara^1^, Satoko Takizawa^3^, Soken Tsuchiya^1^, Gozoh Tsujimoto^4^, Shinji Uemoto^2^, Kazuharu Shimizu^1^

Affiliation

1: Department of Nanobio Drug Discovery, Graduate School of Pharmaceutical Sciences, Kyoto University, Kyoto, Japan.

2: Department of Hepato-Biliary-Pancreatic Surgery & Transplantation, Kyoto University Hospital, Kyoto, Japan.

3: New Frontiers Research Laboratories, Toray Industries Inc., Kanagawa, Japan.

4: Department of Pharmacogenomics, Graduate School of Pharmaceutical Sciences, Kyoto University, Kyoto, Japan.

**Analytical procedures**

**1. Dataset preparation**

The clinical dataset consisted of the 63 clinicopathological information points listed in Table S1. In total, 146 microRNA expression datasheets of both tumor and non-tumor tissues were obtained from the 73 patients. The microRNA expression data was log2 transformed, and normalized by a quantile normalization method [1]. Next, the microRNA expression datasheets of the tumor and non-tumor tissues, and the T/N ratio was concatenated. Since Toray's microRNA microarray chip (the miRBase version 12) contains 866 human microRNA probes, the generated master datasheet consisted of 73 rows and 2598 (=866×3) columns.

**2. Filtering of variables**

In general, poorly expressed microRNAs tend to have a less repressive impact on biological functions, as compared with highly expressed microRNAs. Therefore, the low expressed microRNAs throughout the samples were excluded from this analysis. The filtering criteria were as follows; if the 75%tile values of a log2 expression of a microRNA did not exceed 6 both in tumor and non-tumor samples, then the microRNA was excluded.

**3. Overview of whole microRNA expression profile and unsupervised clustergram.**

Supplementary Figure 1 illustrates a heatmap of the microRNA expression in the HCC tumor and non-tumor tissue specimens. The tumor-derived microRNAs profile and non-tumor derived microRNAs profile are almost separated. This finding indicates that this microRNA profiling techniques is working properly in this study.

**4. Construction and validation of recurrence-free survival prediction models**

A flow chart of the prediction model construction and validation is illustrated in Figure 1. We used a leave-one-out closs-validation method. In each leave-one-out cycle, one patient data was separated from the dataset as test data, and the remaining data were used as a training dataset. Individual microRNA datasets or principal component analysis datasets were the subjects for the next univariate Cox proportional hazard model. According to p-values of the univariate Cox analysis, individual microRNAs or principal components (PC) were prioritized. Using a different number of variables (ranging 1 to 30 variables), a multivariate Cox proportional hazard model was trained. Next, the predicted hazard ratio and predicted survival curve were calculated using the saved test data and a baseline survival curve of the training dataset. The best predictive model was determined by time-averaged AUROC, as described below.

**5. Time-averaged AUROCs**

The Cox proportional hazard model can analyze data containing censored cases, and can predict survival curves of test cases using a baseline survival curve and predicted hazard ratios. However, it is difficult to evaluate the prediction accuracy of this Cox proportional hazard model. Usually, analysts stratify patients according to Cox model predictions and compare difference between the groups using generalized-Wilcoxon or log-rank tests, or calculate the sensitivity, specificity, or area under the Receiver Operator Characteristic curve (AUROC) at a given time point, such as the 2-year survival, using the predictions and outcomes of patients relevant for the time point. However, the prediction efficiency of these analyses depend upon the stratification method of the patients and the selection of a prediction time point. Therefore, we proposed a new evaluation index for the survival prediction efficiency of the Cox proportional hazard model, i.e. the time-averaged AUROC. The time-averaged AUROC (
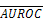
) is defined as below:


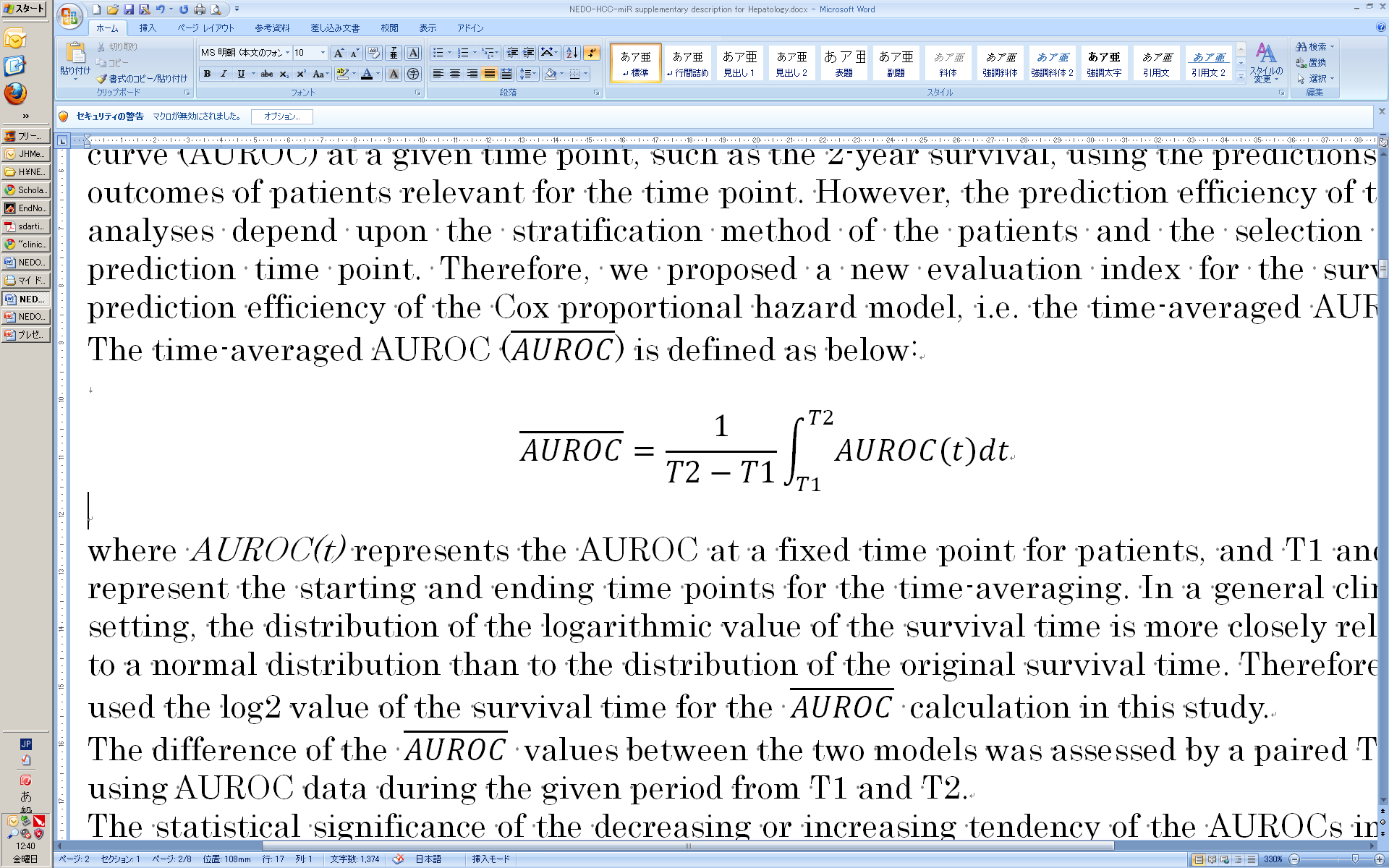


where *AUROC(t)* represents the AUROC at a fixed time point for patients, and T1 and T2 represent the starting and ending time points for the time-averaging. In a general clinical setting, the distribution of the logarithmic value of the survival time is more closely related to a normal distribution than to the distribution of the original survival time. Therefore, we used the log2 value of the survival time for the
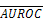
 calculation in this study.

The difference of the
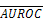
 values between the two models was assessed by a paired T-test using AUROC data during the given period from T1 and T2.

The statistical significance of the decreasing or increasing tendency of the AUROCs in the given time period was assessed by Pearson's correlation analysis.

**6. Permutation analysis and calculation of false discovery rate.**

Table 2 lists HCC recurrence-related microRNAs. To generate this table, we repeated univariate Cox proportional hazard model analysis 386 times (=193 T-miRs + 193 N-miRs). In the case of a multiple-testing procedure, results with low p-values can be obtained by chance. Therefore, we estimated false discovery rate (FDR) by permutation analysis technique. Outcome dataset (consisting of recurrence-free survival and censoring information) was permutated randomly. Using this permutated outcome data and original microRNA expression dataset, we performed univariate Cox proportional hazard model analysis for all 193 T- and 193 N-miRs. We repeated this permutated analysis 1000 times, and obtained a set (S*_i_*) of 1000 p-values for each i-th ranked microRNA (i = 1~193). Then, the FDR of i-th ranked microRNA was defined as (number of p-values in the S*_i_* < the original p-value of the i-th ranked microRNA)/1000.

**7. Contribution analysis of each microRNA in the Cox proportional hazard model with principal component analysis (PCA) data**

In the Cox analysis using PCA data, the
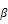
 values for each principal component are provided below:


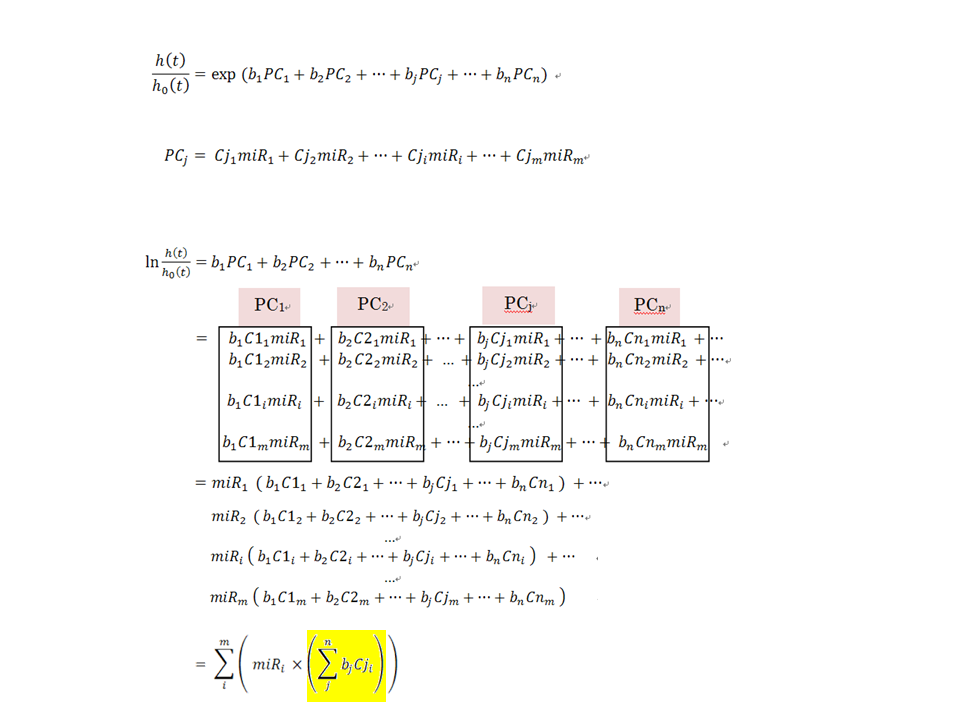


where h(t) and ho(t) represent the hazard at a given time point 't' for subject case and baseline survival curve, respectively, and b_j_ and PC_j_ are the
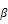
 values of the j-th principal component (PC). On the other hand, each PC can be expressed as:


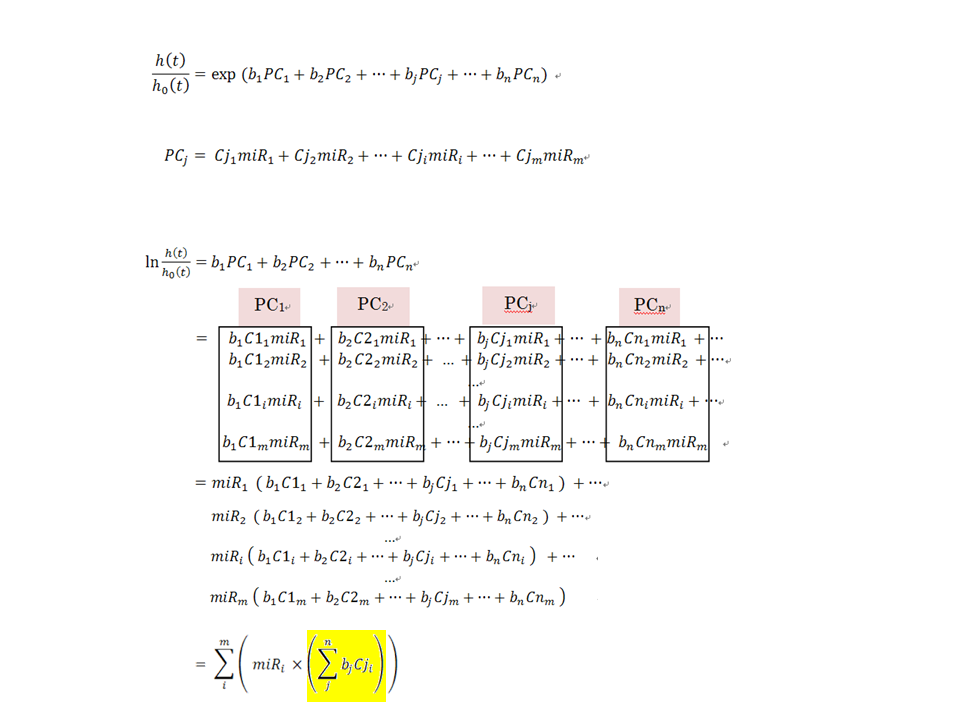


where
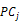
,
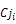
 and
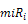
 represent the j-th PC, the coefficient of the i-th microRNA in the j-th PC, and the centered expression values of the i-th microRNA, respectively.

Thus, the natural logarithmic value of hazard ratio can be expressed as below:


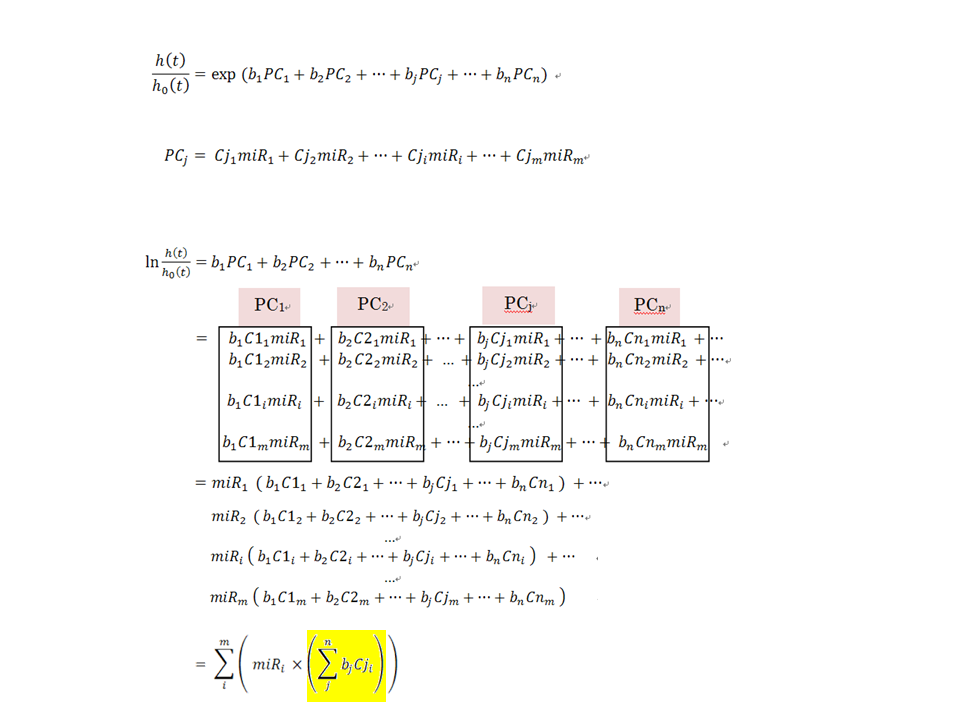


where m and n represent the number of microRNA and PCs.

Therefore, the contribution of the i-th microRNA can be expressed as:
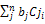
.

To estimate 95% confidence interval (CI) of these coefficients, we utilized a case-resampling bootstrap technique. In the construction procedures of the prediction model, a resampled dataset was generated from the PCA data, and coefficients,
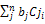
, were calculated using the resampled dataset. This bootstrap step were repeated 1000 times. The 95% CI was defined as an interval between 2.5 and 97.5 percentile of 1000 coefficients generated by this bootstrap resampling procedure.

**8. mRNA expression profiling by DNA chip**

For the DNA microarray analysis, 400 ng of total RNA was amplified and labeled using an TargetAmp™ 1-Round Aminoallyl-aRNA Amplification Kit (Epicentre, Madison, WI) according to the manufacturer’s instructions. Each non-tumor sample of aRNA labeled with Cy3 and tumor aRNA labeled with Cy5 were cohybridized with 3D-Gene™ Human 25K (Toray Industries, Inc., Tokyo, Japan) at 37℃ for 16 h. After hybridization, each DNA chip was washed and dried in an ozone-free booth. Hybridization signals derived from Cy3 and Cy5 were scanned using Scan Array Express (PerkinElmer, Waltham, MA). The scanned image was analyzed using GenePix Pro (Molecular Devices, Sunnyvale, CA). All the analyzed data were scaled by quantile normalization.

**9. Screening and ontology information of putative target genes of miR-96**

MicroRNAs regulate gene expression post-transcriptionally by repressing translation of protein synthesis and inducing degradation of target mRNA. Therefore, the expression level of mRNA regulated by the latter mechanism is supposed to be inversely correlated with the microRNA expression level. In order to identify the putative target genes of miR-96, we screened mRNAs that are inversely correlated to miR-96 expression level in 146 samples (tumor and non-tumor paired samples derived from 73 patients), and that have microRNA target sites predicted by Target Scan v.5.1 (http://www.targetscan.org/) in their 3'-UTR. A list of putative miR-96 with p-values less than 0.01 are shown in Table S9-12. In addition, ontology information of each screened target gene is retrieved from the NCBI Entrez site (http://www.ncbi.nlm.nih.gov/gene), and provided in Table S9-12. We provide mRNA expression dataset consisting of 787 mRNAs that are identified as putative target genes of miR-96 by the Target Scan, in a Microsoft Excel file format (Table S13).

References

1. Bolstad BM, Irizarry RA, Astrand M, Speed TP (2003) A comparison of normalization methods for high density oligonucleotide array data based on variance and bias. Bioinformatics 19: 185-193.
