# Supplementary figures and images for: MicroRNA Profile Predicts Recurrence after Resection in Patients with Hepatocellular Carcinoma within the Milan Criteria
Source: PLoS One. 2011 Jan 27;6(1):e16435. doi: 10.1371/journal.pone.0016435 (PMC3029327; doi:10.1371/journal.pone.0016435)

Figure S1


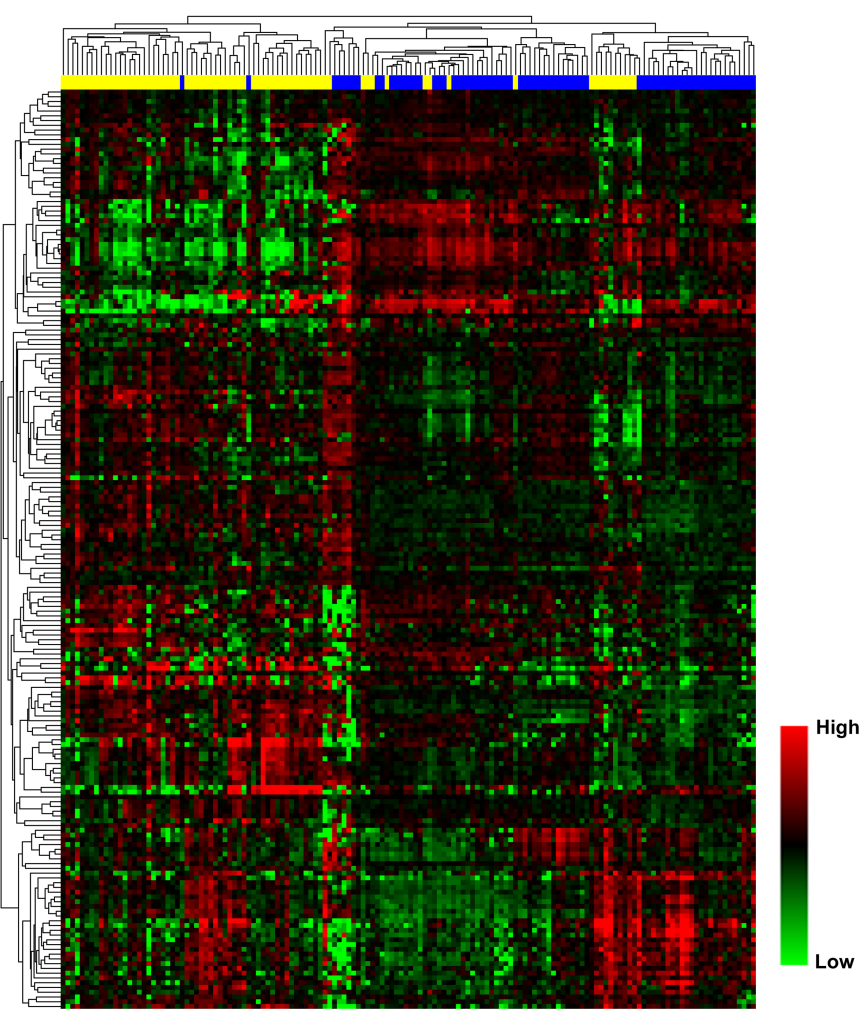

Supplement: Figure S1 — A heatmap and unsupervised clustergram of microRNA expression in tumor and non-tumor tissues of HCC patients. This heatmap represents an overview of microRNA expression profile. The microRNA expression data was centered by 2 directions (i.e., by genes and patients). Red, green, and black represent high, low, and intermediate microRNA expression. Blue and yellow bars on the top of the heatmap represent non-tumor, and tumor tissues. (DOC) [file pone.0016435.s002.doc]

Figure S2


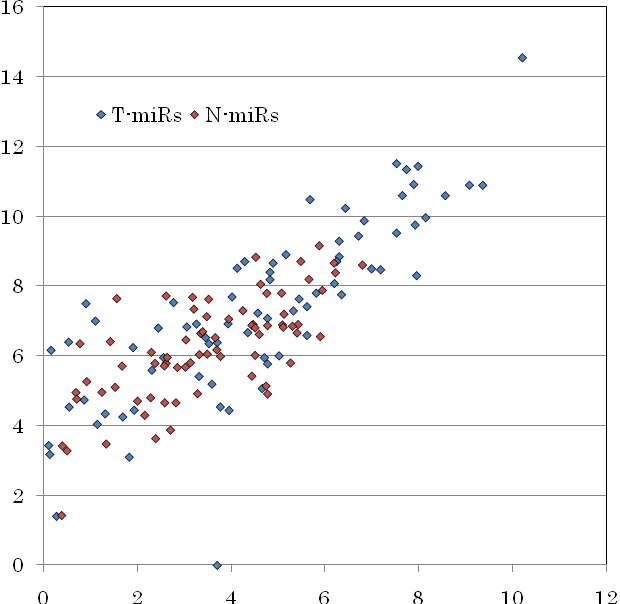

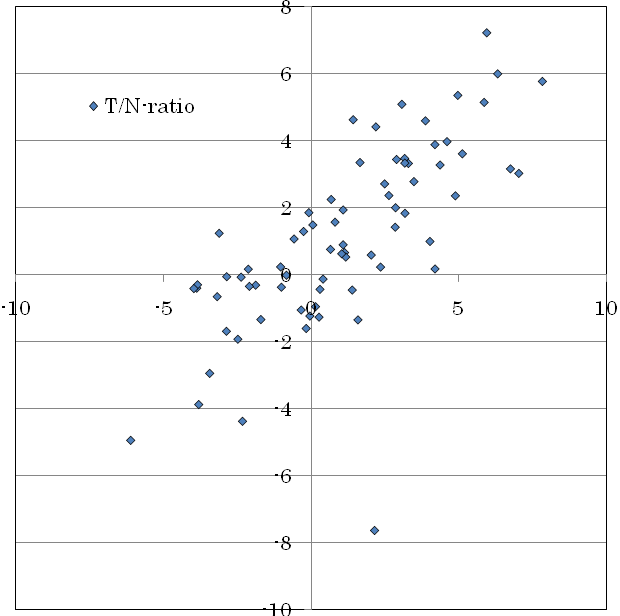


Pearson's Correlation: p<0.0001

Pearson's Correlation: p<0.0001

Supplement: Figure S2 — Correlation between DNA chip and Taqman data for miR-96 expression. DNA chip expression data for miR-96 were validated by Taqman microRNA assay. DNAchip data and Taqman data were significantly correlated (p<0.0001). Left: x-axis: DNA chip data in a log2 scale, y-axis: Taqman data in an arbitrary log2 scale. Right: x-axis: DNA chip data in a log2 scale, y-axis: Taqman data in a log2 scale. (DOC) [file pone.0016435.s003.doc]
