# Supplementary material for: MicroRNA Profile Predicts Recurrence after Resection in Patients with Hepatocellular Carcinoma within the Milan Criteria
Source: PLoS One. 2011 Jan 27;6(1):e16435. doi: 10.1371/journal.pone.0016435 (PMC3029327; doi:10.1371/journal.pone.0016435)
Supplement: Table S1 — Variables in clinicopathological dataset. (DOC) [file pone.0016435.s004.doc]

Table S1

| Variables in clinicopathological dataset (n=63) | | | |
| --- | --- | --- | --- |
| 1 | age | 33 | invasion to serosa* |
| 2 | gender | 34 | no co-existing liver disease** |
| 3 | ICG R15 | 35 | co-existing liver disease (chronic hepatitis)**§ |
| 4 | ICG k | 36 | co-existing liver disease (liver cirrhosis)**§ |
| 5 | clinical stage* | 37 | co-existing liver disease (others)**§ |
| 6 | Child-Pugh points | 38 | T-factor* |
| 7 | Child-Pugh classification | 39 | Hr-factor (hepatic resection)* |
| 8 | serum, AST | 40 | resected other organs |
| 9 | serum, ALT | 41 | curability |
| 10 | blood, platelet | 42 | macroscopic morphology |
| 11 | serum, total-bilirubin | 43 | capsule formation |
| 12 | serum, direct-bilirubin | 44 | infiltration into capsule |
| 13 | serum, albmin | 45 | septum formation |
| 14 | prothrombin activity | 46 | intrahepatic metastasis |
| 15 | prothrombin time | 47 | vessel invasion |
| 16 | alpha feto protein (log10 transformed) | 48 | surgical mergin |
| 17 | PIVKA (log10 transformed) | 49 | postoperative complication |
| 18 | hepatitis B Virus Antigen | 50 | # of tumor¶ |
| 19 | hapatitis C Virus Antibody | 51 | capsule formation ¶ |
| 20 | intraoperative hemorrhage | 52 | infiltration into capsule ¶ |
| 21 | operation time | 53 | septum formation ¶ |
| 22 | multiplicity of tumor | 54 | vessel invasion ¶ |
| 23 | # of tumors § | 55 | intrahepatic metastasis ¶ |
| 24 | longest diameter of tumor§ | 56 | pathological tumor grade |
| 25 | length of minor axis of tumor § | 57 | co-existing liver disease (chronic hepatitis)**¶ |
| 26 | tumor location (anterior segment)** | 58 | co-existing liver disease (liver cirrhosis)**¶ |
| 27 | tumor location (posterior segment)** | 59 | co-existing liver disease (others)**¶ |
| 28 | tumor location (medial segment)** | 60 | grade of chronic hepatitis ¶† |
| 29 | tumor location (lateral segment)** | 61 | stage of chronic hepatitis ¶† |
| 30 | tumor location (caudate segment)** | 62 | T-facor*¶ |
| 31 | longest diameter of resected tumor | 63 | curability*¶ |
| 32 | H-factor* |  |  |

*: the 4th Japanese Guideline for HCC[1]

**: dummy variables

§:clinical diagnosis

¶:pathological diagnosis

†: new Inuyama classification (1996)[2]

1. Liver Cancer Study Group of Japan a (2000) The General Rules for the Clinical and Pathological Study of Primary Liver Cancer. Tokyo: Kanehara & Co., Ltd.

2. Ichida F, Tsuji T, Omata M, Ichida T, Inoue K, et al. (1996) New Inuyama Classification; new criteria for histological assessment of chronic hepatitis. International Hepatology Communications 6: 112-119.
