# Supplementary material for: MicroRNA Profile Predicts Recurrence after Resection in Patients with Hepatocellular Carcinoma within the Milan Criteria
Source: PLoS One. 2011 Jan 27;6(1):e16435. doi: 10.1371/journal.pone.0016435 (PMC3029327; doi:10.1371/journal.pone.0016435)
Supplement: Table S2 — Putative miR-96 target genes which expression is inversely correlated with miR-96 expression. *: these values are provided by TargetScan v.5.1, **: rank of total context score among 787 putative miR-96 target genes predicted by TargetScan. †: Pearson's correlation coefficients and p-values, ‡: Pubmed hit count using key words of gene symbol and “HCC, liver cancer, hepatocellular carcinoma”, accessed on Dec 26, 2010. (DOC) [file pone.0016435.s005.doc]

| **Target gene** | **Gene name** | **context score*** | **Aggregate P_CT_*** | **Rank**** | **correlation coefficient†** | **p-value†** | **Gene Ontology function** | **Gene Ontology Process** | **Pubmed  hit count‡** |
| --- | --- | --- | --- | --- | --- | --- | --- | --- | --- |
| TAF15 | TAF15 RNA polymerase II, TATA box binding protein (TBP)-associated factor | -0.24 | 0.73 | 337 | -0.3977 | 0.000001 | nucleotide binding |  | 0 |
| LRP6 | low density lipoprotein receptor-related protein 6 | -0.2 | 0.74 | 419 | -0.3872 | 0.000001 | low-density lipoprotein receptor activity Wnt-protein binding | negative regulation of Wnt receptor signaling pathway | 4 |
| FOXO1 | forkhead box O1 | -0.6 | 0.95 | 28 | -0.3347 | 0.000036 | sequence-specific DNA binding transcription factor activity | **regulation of cell proliferation** | **43** |
| ACADSB | acyl-Coenzyme A dehydrogenase, short/branched chain | -0.1 | 0.69 | 675 | -0.3331 | 0.000040 | acyl-CoA dehydrogenase activity | lipid metabolic process | 0 |
| MAP2K1 | mitogen-activated protein kinase kinase 1 | -0.23 | 0.8 | 354 | -0.3317 | 0.000043 | MAP kinase kinase activity Ras GTPase binding | **cell motility negative regulation of cell proliferation** | **22** |
| INTS6 | integrator complex subunit 6 | -0.21 | 0.74 | 395 | -0.3132 | 0.000118 | transmembrane receptor activity | snRNA processing | 1 |
| TAPT1 | transmembrane anterior posterior transformation 1 | -0.21 | 0.74 | 395 | -0.2774 | 0.000701 | growth hormone-releasing hormone receptor activity | embryonic skeletal system development | 0 |
| EPB41L4B | erythrocyte membrane protein band 4.1 like 4B | -0.2 | 0.83 | 419 | -0.2733 | 0.000844 | cytoskeletal protein binding |  | 0 |
| CADM2 | cell adhesion molecule 2 | -0.16 | 0.69 | 552 | -0.2686 | 0.001047 |  |  | 0 |
| SEC14L2 | SEC14-like 2 (S. cerevisiae) | -0.22 | 0.59 | 374 | -0.2680 | 0.001072 | phospholipid binding vitamin E binding | positive regulation of cholesterol biosynthetic process | 6 |
| UNC13C | unc-13 homolog C (C. elegans) | -0.8 | 0.94 | 6 | -0.2679 | 0.001076 | metal ion binding | Exocytosis, synaptic transmission | 0 |
| CREB3L2 | cAMP responsive element binding protein 3-like 2 | -0.42 | 0.71 | 119 | -0.2667 | 0.001139 | cAMP response element binding transcription factor activity |  | 0 |
| ETNK2 | ethanolamine kinase 2 | -0.08 | 0.74 | 714 | -0.2662 | 0.001164 | ethanolamine kinase activity | placenta development | 0 |
| PIK3R1 | phosphoinositide-3-kinase, regulatory subunit 1 (alpha) | -0.28 | 0.74 | 259 | -0.2655 | 0.001201 | ErbB-3 class receptor binding phosphoinositide 3-kinase regulator activity | positive regulation of glucose import insulin receptor signaling pathway | 1 |
| CLIC5 | chloride intracellular channel 5 | -0.05 | 0.74 | 745 | -0.2517 | 0.002177 | voltage-gated chloride channel activity | chloride transport | 0 |
| ELL2 | elongation factor, RNA polymerase II, 2 | -0.17 | 0.74 | 510 | -0.2497 | 0.002369 | RNA polymerase II transcription elongation factor activity | regulation of transcription | 0 |
| EGR3 | early growth response 3 | -0.17 | 0.74 | 510 | -0.2493 | 0.002414 | sequence-specific DNA binding transcription factor activity | circadian rhythm peripheral nervous system development | 1 |
| GPM6A | glycoprotein M6A | -0.27 | 0.6 | 276 | -0.2486 | 0.002482 | calcium channel activity |  | 0 |
| REV1 | REV1 homolog (S. cerevisiae) | -0.52 | 0.95 | 50 | -0.2481 | 0.002530 | damaged DNA binding | DNA repair | 0 |
| SLC1A2 | solute carrier family 1 (glial high affinity glutamate transporter), member 2 | -0.32 | 0.74 | 210 | -0.2461 | 0.002753 | L-glutamate transmembrane transporter activity | synaptic transmission | 0 |
| LUZP1 | leucine zipper protein 1 | -0.21 | 0.81 | 395 | -0.2440 | 0.002994 |  |  | 0 |
| TMEM145 | transmembrane protein 145 | -0.17 | 0.74 | 510 | -0.2431 | 0.003110 |  |  | 0 |
| GPHN | gephyrin | -0.35 | 0.66 | 172 | -0.2344 | 0.004410 | nucleotidyltransferase activity | Mo-molybdopterin cofactor biosynthetic process | 0 |
| ERLIN1 | ER lipid raft associated 1 | -0.35 | 0.72 | 172 | -0.2325 | 0.004751 |  | ER-associated protein catabolic process | 0 |
| ANKRD52 | ankyrin repeat domain 52 | -0.25 | 0.86 | 311 | -0.2322 | 0.004795 |  |  | 0 |
| CUL4A | cullin 4A | -0.04 | 0.72 | 755 | -0.2300 | 0.005233 | ubiquitin protein ligase binding | DNA repair, cell cycle arrest | 2 |
| FAM13A1 | family with sequence similarity 13, member A1 | -0.17 | 0.7 | 510 | -0.2276 | 0.005729 | GTPase activator activity | signal transduction | 0 |
| SCML4 | sex comb on midleg-like 4 (Drosophila) | -0.21 | 0.66 | 395 | -0.2252 | 0.006270 | DNA binding | regulation of transcription | 0 |
| SYT9 | synaptotagmin IX | -0.22 | 0.28 | 374 | -0.2241 | 0.006544 | transporter activity | regulation of calcium ion-dependent exocytosis, regulation of insulin secretion | 0 |
| IRS1 | insulin receptor substrate 1 | -0.24 | 0.61 | 337 | -0.2229 | 0.006841 | insulin-like growth factor receptor binding phosphoinositide 3-kinase binding | insulin-like growth factor receptor signaling pathway, PI3-kinase cascade, positive regulation of cell proliferation | 92 |
| TSKU | tsukushin | -0.26 | 0.79 | 294 | -0.2227 | 0.006893 | protein binding |  | 0 |
| SMCR8 | Smith-Magenis syndrome chromosome region, candidate 8 | -0.12 | 0.74 | 636 | -0.2197 | 0.007719 |  |  | 0 |
| GJA3 | gap junction protein, alpha 3, 46kDa | -0.16 | 0.74 | 552 | -0.2181 | 0.008172 | gap junction channel activity | cell-cell signaling | 0 |

Table S2
