# Supplementary material for: MicroRNA Profile Predicts Recurrence after Resection in Patients with Hepatocellular Carcinoma within the Milan Criteria
Source: PLoS One. 2011 Jan 27;6(1):e16435. doi: 10.1371/journal.pone.0016435 (PMC3029327; doi:10.1371/journal.pone.0016435)
Supplement: Table S3 — Significantly up-regulated microRNAs in HCC tumor tissues compared to non-tumor tissues. Up-regulated microRNAs with p<0.01 are listed. T-miRs, N-miRs: mean values of each T-miR and NmiR expression in log2 scale, fold change: expression ratio of each T-miR compared with corresponding N-miR, p-value: p-values of paired T-test. Order of microRNA is sorted by fold-change. (DOC) [file pone.0016435.s006.doc]

Table S3

| miR name | T-miRs | N-miRs | fold-change | p-value |
| --- | --- | --- | --- | --- |
| miR-224 | 7.1005 | 5.8716 | 2.3439 | 0.00006 |
| miR-221 | 8.9564 | 7.7724 | 2.2721 | <0.00001 |
| miR-96 | 4.5990 | 3.4966 | 2.1470 | 0.00302 |
| miR-130b | 6.6031 | 5.5227 | 2.1146 | <0.00001 |
| miR-452 | 5.7578 | 4.9143 | 1.7944 | 0.00259 |
| miR-106b | 9.8617 | 9.0395 | 1.7681 | <0.00001 |
| miR-222 | 7.3771 | 6.5574 | 1.7651 | <0.00001 |
| miR-21 | 12.0351 | 11.2189 | 1.7608 | <0.00001 |
| miR-18b | 5.6000 | 4.8291 | 1.7064 | 0.00248 |
| miR-93 | 8.6849 | 7.9448 | 1.6703 | <0.00001 |
| miR-425 | 8.0632 | 7.3283 | 1.6643 | <0.00001 |
| miR-1268 | 8.7846 | 8.0725 | 1.6381 | <0.00001 |
| miR-675 | 6.0331 | 5.3300 | 1.6280 | 0.00033 |
| miR-1469 | 9.1517 | 8.4493 | 1.6272 | 0.00001 |
| miR-1228* | 9.7963 | 9.1177 | 1.6006 | <0.00001 |
| miR-18a | 5.9948 | 5.3167 | 1.6001 | 0.00841 |
| miR-664 | 8.2808 | 7.6315 | 1.5684 | <0.00001 |
| miR-1915 | 7.3535 | 6.7203 | 1.5510 | 0.00016 |
| miR-362-3p | 5.4439 | 4.8225 | 1.5384 | 0.00398 |
| miR-301a | 6.7046 | 6.0914 | 1.5297 | 0.00444 |
| miR-663 | 9.8008 | 9.1948 | 1.5221 | 0.00066 |
| miR-34a | 9.6314 | 9.0273 | 1.5201 | 0.00001 |
| miR-660 | 6.8796 | 6.2846 | 1.5104 | 0.00016 |
| miR-193b | 8.9301 | 8.3360 | 1.5095 | 0.00014 |
| miR-151-5p | 8.9698 | 8.4085 | 1.4756 | 0.00003 |
| miR-25 | 8.9278 | 8.3846 | 1.4572 | <0.00001 |
| miR-1909 | 7.1333 | 6.5913 | 1.4560 | 0.00001 |
| miR-494 | 9.7928 | 9.3206 | 1.3872 | 0.00023 |
| miR-638 | 10.4487 | 9.9916 | 1.3728 | 0.00023 |
| miR-17 | 9.8009 | 9.3496 | 1.3673 | 0.00176 |
| miR-107 | 10.9199 | 10.5205 | 1.3190 | <0.00001 |
| miR-365 | 8.1769 | 7.7796 | 1.3170 | 0.00150 |
| miR-151-3p | 7.1208 | 6.7503 | 1.2928 | 0.00196 |
| miR-191 | 10.3609 | 9.9908 | 1.2924 | <0.00001 |
| miR-103 | 11.1865 | 10.8314 | 1.2791 | 0.00059 |
| miR-140-3p | 8.3389 | 8.0014 | 1.2635 | 0.00015 |
| miR-185 | 7.7581 | 7.4236 | 1.2609 | 0.00318 |
| miR-361-5p | 8.0541 | 7.7214 | 1.2594 | 0.00004 |
| miR-15a | 8.7356 | 8.4428 | 1.2250 | 0.00068 |
| miR-320a | 8.1341 | 7.8845 | 1.1888 | 0.00792 |
| miR-29a | 11.1116 | 10.9187 | 1.1430 | 0.00739 |
| miR-1826 | 14.6080 | 14.4679 | 1.1020 | 0.00303 |
