# Supplementary material for: MicroRNA Profile Predicts Recurrence after Resection in Patients with Hepatocellular Carcinoma within the Milan Criteria
Source: PLoS One. 2011 Jan 27;6(1):e16435. doi: 10.1371/journal.pone.0016435 (PMC3029327; doi:10.1371/journal.pone.0016435)
Supplement: Table S4 — Significantly down-regulated microRNAs in HCC tumor tissues compared to non-tumor tissues. Down-regulated microRNAs with p<0.01 are listed. T-miRs, N-miRs: mean values of each T-miR and NmiR expression in log2 scale, fold change: expression ratio of each T-miR compared with corresponding N-miR, p-value: p-values of paired T-test. Order of microRNA is sorted by fold-change. (DOC) [file pone.0016435.s007.doc]

Table S4

| miR name | T-miR | N-miR | fold-change | p-value |
| --- | --- | --- | --- | --- |
| miR-200a | 5.8183 | 8.5496 | 0.1506 | <0.00001 |
| miR-375 | 3.7664 | 6.0955 | 0.1990 | <0.00001 |
| miR-200b | 6.7698 | 9.0919 | 0.2000 | <0.00001 |
| miR-199b-3p | 8.6670 | 10.8304 | 0.2232 | <0.00001 |
| miR-199a-3p | 8.7558 | 10.8862 | 0.2284 | <0.00001 |
| miR-199a-5p | 8.5426 | 10.5858 | 0.2426 | <0.00001 |
| miR-150 | 6.0219 | 7.7215 | 0.3079 | <0.00001 |
| miR-10a | 6.8784 | 8.4695 | 0.3319 | <0.00001 |
| miR-424 | 6.8159 | 8.3325 | 0.3495 | <0.00001 |
| miR-214 | 6.9813 | 8.4080 | 0.3720 | <0.00001 |
| miR-139-5p | 4.8902 | 6.3078 | 0.3744 | <0.00001 |
| miR-451 | 11.4110 | 12.7522 | 0.3947 | <0.00001 |
| miR-142-3p | 4.3882 | 5.5899 | 0.4348 | 0.00001 |
| miR-142-5p | 6.7147 | 7.8031 | 0.4703 | 0.00003 |
| miR-223 | 8.3237 | 9.3963 | 0.4755 | <0.00001 |
| miR-146a | 6.4679 | 7.5048 | 0.4874 | 0.00001 |
| miR-486-5p | 4.9461 | 5.9425 | 0.5012 | <0.00001 |
| miR-30a* | 6.1348 | 7.1145 | 0.5071 | <0.00001 |
| miR-130a | 8.2858 | 9.2523 | 0.5118 | <0.00001 |
| miR-376c | 4.9713 | 5.8816 | 0.5321 | 0.00542 |
| miR-378 | 8.3006 | 9.1927 | 0.5388 | <0.00001 |
| miR-125a-5p | 8.1841 | 9.0551 | 0.5468 | <0.00001 |
| miR-195 | 8.8553 | 9.7022 | 0.5560 | <0.00001 |
| miR-497 | 7.3460 | 8.1444 | 0.5750 | 0.00002 |
| miR-422a | 7.1003 | 7.8778 | 0.5834 | <0.00001 |
| miR-342-3p | 8.0099 | 8.6998 | 0.6199 | <0.00001 |
| miR-125b | 10.3309 | 10.9920 | 0.6324 | 0.00001 |
| miR-101 | 6.4174 | 7.0780 | 0.6326 | 0.00005 |
| miR-1249 | 4.6112 | 5.2266 | 0.6527 | 0.00728 |
| miR-30e* | 7.7371 | 8.3455 | 0.6559 | <0.00001 |
| miR-1238 | 5.1302 | 5.7055 | 0.6711 | 0.00216 |
| miR-335 | 6.6345 | 7.1763 | 0.6869 | 0.00812 |
| miR-145 | 9.6902 | 10.2310 | 0.6874 | 0.00037 |
| miR-455-5p | 6.0197 | 6.5470 | 0.6939 | 0.00087 |
| miR-22* | 5.4614 | 5.9578 | 0.7089 | 0.00632 |
| miR-99a | 10.4387 | 10.9317 | 0.7105 | 0.00047 |
| miR-146b-5p | 9.6150 | 10.0391 | 0.7453 | 0.00990 |
| miR-100 | 10.4659 | 10.8843 | 0.7482 | 0.00039 |
| miR-143 | 10.8380 | 11.2255 | 0.7645 | 0.00411 |
| let-7g | 11.0311 | 11.4135 | 0.7672 | 0.00078 |
| miR-181a | 7.5643 | 7.9462 | 0.7674 | 0.00515 |
| miR-99b | 7.5207 | 7.8866 | 0.7760 | 0.00058 |
| miR-22 | 12.1227 | 12.4560 | 0.7937 | 0.00006 |
| miR-30a | 10.4827 | 10.8113 | 0.7963 | 0.00034 |
| miR-26a | 12.3555 | 12.6796 | 0.7988 | 0.00004 |
| miR-30c | 10.9891 | 11.2535 | 0.8325 | 0.00007 |
| let-7c | 11.5850 | 11.8016 | 0.8606 | 0.00070 |
| let-7a | 12.2072 | 12.3928 | 0.8793 | 0.00395 |
| miR-1280 | 14.0985 | 14.2756 | 0.8845 | 0.00168 |
