# Supplementary material for: MicroRNA Profile Predicts Recurrence after Resection in Patients with Hepatocellular Carcinoma within the Milan Criteria
Source: PLoS One. 2011 Jan 27;6(1):e16435. doi: 10.1371/journal.pone.0016435 (PMC3029327; doi:10.1371/journal.pone.0016435)
Supplement: Table S5 — Differentially expressed microRNA compared with normal liver tissues. Differentially expressed microRNAs with p<0.05 are listed. T-miRs, N-miRs: mean values of each T-miR and NmiR expression in log2 scale, fold change: expression ratio of each T-miR or N-miR compared with normal liver tissues (n = 4), p-value: p-values of unpaired T-test. The miR order is sorted by fold-change. (DOC) [file pone.0016435.s008.doc]

Table S5

|  | miR name | T-miRs | normal | fold-change | p-value |  | miR name | N-miRs | normal | fold-change | p-value |
| --- | --- | --- | --- | --- | --- | --- | --- | --- | --- | --- | --- |
| Up-regulated miRs | | | | | | | | | | | |
|  | miR-96 | 4.5990 | 1.8477 | 6.7330 | 0.03489 |  | miR-96 | 3.4966 | 1.8477 | 3.1359 | 0.04947 |
|  | miR-222 | 7.3771 | 5.1257 | 4.7614 | 0.00001 |  | miR-222 | 6.5574 | 5.1257 | 2.6976 | 0.00939 |
|  | miR-18b | 5.6000 | 3.4268 | 4.5102 | 0.03660 |  | miR-886-3p | 7.7848 | 6.5724 | 2.3173 | 0.04503 |
|  | miR-224 | 7.1005 | 5.1620 | 3.8330 | 0.04204 |  | miR-146b-5p | 10.0391 | 9.0101 | 2.0406 | 0.00038 |
|  | miR-221 | 8.9564 | 7.3352 | 3.0763 | 0.00010 |  | miR-199a-3p | 10.8862 | 10.1048 | 1.7188 | 0.01696 |
|  | miR-21 | 12.0351 | 10.4628 | 2.9737 | 0.00007 |  | miR-181a | 7.9462 | 7.1682 | 1.7147 | 0.00651 |
|  | miR-1469 | 9.1517 | 7.7425 | 2.6558 | 0.00302 |  | miR-199a-5p | 10.5858 | 9.8103 | 1.7118 | 0.01517 |
|  | miR-362-3p | 5.4439 | 4.1095 | 2.5217 | 0.04277 |  | miR-199b-3p | 10.8304 | 10.1247 | 1.6309 | 0.02380 |
|  | miR-663 | 9.8008 | 8.5582 | 2.3663 | 0.01236 |  | miR-10a | 8.4695 | 7.7943 | 1.5969 | 0.03236 |
|  | miR-106b | 9.8617 | 8.6820 | 2.2654 | 0.00023 |  | miR-214 | 8.4080 | 7.7728 | 1.5531 | 0.03594 |
|  | miR-34a | 9.6314 | 8.4736 | 2.2312 | 0.01399 |  |  |  |  |  |  |
|  | miR-25 | 8.9278 | 8.2994 | 1.5459 | 0.03904 |  |  |  |  |  |  |
| Down-regulated miRs | | | | | | | | | | | |
|  | miR-375 | 3.7664 | 6.2964 | 0.1731 | 0.01715 |  | miR-1238 | 5.7055 | 7.0204 | 0.4020 | 0.00922 |
|  | miR-1249 | 4.6112 | 6.6075 | 0.2506 | 0.00533 |  | miR-296-5p | 8.4425 | 9.6868 | 0.4221 | 0.01721 |
|  | miR-139-5p | 4.8902 | 6.8648 | 0.2544 | 0.01140 |  | miR-1228 | 6.0568 | 7.1856 | 0.4573 | 0.02378 |
|  | miR-1238 | 5.1302 | 7.0204 | 0.2698 | 0.00990 |  | miR-1913 | 8.3088 | 9.2908 | 0.5063 | 0.00631 |
|  | miR-625* | 5.3830 | 7.0589 | 0.3130 | 0.00344 |  | miR-940 | 6.9842 | 7.8839 | 0.5360 | 0.02220 |
|  | miR-422a | 7.1003 | 8.7371 | 0.3216 | 0.00783 |  | miR-422a | 7.8778 | 8.7371 | 0.5512 | 0.01481 |
|  | miR-486-5p | 4.9461 | 6.4846 | 0.3442 | 0.02208 |  | miR-148a | 10.3720 | 11.1663 | 0.5766 | 0.03234 |
|  | miR-378 | 8.3006 | 9.8241 | 0.3478 | 0.00364 |  | miR-193a-5p | 5.7622 | 6.5466 | 0.5806 | 0.02435 |
|  | miR-296-5p | 8.2098 | 9.6868 | 0.3592 | 0.00125 |  | miR-365 | 7.7796 | 8.5251 | 0.5965 | 0.01594 |
|  | miR-101 | 6.4174 | 7.7477 | 0.3977 | 0.01811 |  | miR-101 | 7.0780 | 7.7477 | 0.6286 | 0.04963 |
|  | miR-1228 | 5.9237 | 7.1856 | 0.4170 | 0.01134 |  | miR-1201 | 9.0958 | 9.7294 | 0.6446 | 0.04848 |
|  | miR-1913 | 8.1404 | 9.2908 | 0.4505 | 0.00105 |  | miR-92a | 8.5022 | 8.9180 | 0.7496 | 0.04124 |
|  | miR-193a-5p | 5.4028 | 6.5466 | 0.4526 | 0.02306 |  |  |  |  |  |  |
|  | miR-30e* | 7.7371 | 8.5683 | 0.5620 | 0.04295 |  |  |  |  |  |  |
|  | miR-125a-3p | 7.5449 | 8.2814 | 0.6002 | 0.04314 |  |  |  |  |  |  |
