# Supplementary material for: MicroRNA Profile Predicts Recurrence after Resection in Patients with Hepatocellular Carcinoma within the Milan Criteria
Source: PLoS One. 2011 Jan 27;6(1):e16435. doi: 10.1371/journal.pone.0016435 (PMC3029327; doi:10.1371/journal.pone.0016435)
Supplement: Table S6 — Differentially expressed microRNAs depending upon HBV status. *: p-values of Student's T-test. Differentially expressed microRNAs with p<0.05 are listed. (DOC) [file pone.0016435.s009.doc]

Table S6

|  | T-miR | |  |  |  |  | N-miR | |  |  |
| --- | --- | --- | --- | --- | --- | --- | --- | --- | --- | --- |
| miR | HBV(+) | HBV(-) | Diff | p-value* |  | miR | HBV(+) | HBV(-) | Diff | p-value* |
| Up-regulated |  |  |  |  |  | Up-regulated |  |  |  |  |
| miR-106b | 10.3206 | 9.7715 | 0.5491 | 0.0034 |  | miR-24 | 11.6927 | 11.5276 | 0.1651 | 0.0435 |
| miR-18a | 7.4176 | 5.7149 | 1.7026 | 0.0053 |  |  |  |  |  |  |
| miR-18b | 6.9740 | 5.3297 | 1.6443 | 0.0082 |  |  |  |  |  |  |
| miR-19b | 12.0658 | 11.4107 | 0.6551 | 0.0126 |  |  |  |  |  |  |
| miR-483-3p | 6.0029 | 3.9654 | 2.0375 | 0.0157 |  |  |  |  |  |  |
| miR-92a | 9.0847 | 8.4392 | 0.6455 | 0.0167 |  |  |  |  |  |  |
| miR-19a | 10.0270 | 9.3212 | 0.7057 | 0.0195 |  |  |  |  |  |  |
| miR-146a | 7.3693 | 6.2906 | 1.0786 | 0.0298 |  |  |  |  |  |  |
| miR-375 | 4.9232 | 3.5388 | 1.3844 | 0.0320 |  |  |  |  |  |  |
| miR-483-5p | 6.3649 | 4.7003 | 1.6646 | 0.0368 |  |  |  |  |  |  |
| miR-25 | 9.2463 | 8.8651 | 0.3812 | 0.0408 |  |  |  |  |  |  |
| miR-320a | 8.5294 | 8.0563 | 0.4731 | 0.0484 |  |  |  |  |  |  |
| miR-96 | 5.9103 | 4.3410 | 1.5692 | 0.0494 |  |  |  |  |  |  |
| Down-regulated | |  |  |  |  | Down-regulated | |  |  |  |
| miR-34b* | 4.7227 | 6.3285 | -1.6058 | 0.0001 |  | miR-204 | 5.0377 | 5.8588 | -0.8211 | 0.0035 |
| miR-99b | 6.8002 | 7.6624 | -0.8622 | 0.0006 |  | miR-186 | 4.9359 | 5.8206 | -0.8847 | 0.0045 |
| miR-29b | 8.9477 | 9.5701 | -0.6223 | 0.0018 |  | miR-320a | 7.5878 | 7.9429 | -0.3550 | 0.0047 |
| miR-125b | 9.3161 | 10.5306 | -1.2145 | 0.0019 |  | miR-335 | 6.3978 | 7.3294 | -0.9316 | 0.0106 |
| miR-99a | 9.6034 | 10.6030 | -0.9996 | 0.0024 |  | miR-486-5p | 5.3699 | 6.0552 | -0.6853 | 0.0121 |
| miR-100 | 9.7506 | 10.6066 | -0.8560 | 0.0029 |  | miR-320b | 7.6168 | 7.8877 | -0.2709 | 0.0215 |
| miR-34a | 8.9715 | 9.7613 | -0.7898 | 0.0052 |  | miR-101 | 6.6977 | 7.1528 | -0.4551 | 0.0282 |
| miR-365 | 7.4817 | 8.3136 | -0.8319 | 0.0052 |  | miR-374a | 5.2447 | 5.8441 | -0.5994 | 0.0294 |
| miR-30e* | 7.1643 | 7.8498 | -0.6855 | 0.0059 |  | miR-361-5p | 7.5175 | 7.7615 | -0.2439 | 0.0305 |
| miR-29c | 8.8776 | 9.5295 | -0.6519 | 0.0102 |  | miR-30e* | 8.0976 | 8.3943 | -0.2967 | 0.0326 |
| miR-193b | 8.1940 | 9.0749 | -0.8809 | 0.0139 |  | miR-28-5p | 7.9896 | 8.5349 | -0.5453 | 0.0366 |
| miR-30a | 10.0684 | 10.5642 | -0.4958 | 0.0156 |  | miR-20a | 8.4863 | 9.2370 | -0.7506 | 0.0381 |
| miR-374b | 4.2973 | 5.4034 | -1.1061 | 0.0201 |  | miR-452 | 4.2277 | 5.0494 | -0.8217 | 0.0405 |
| miR-195 | 8.1499 | 8.9941 | -0.8442 | 0.0232 |  | miR-126* | 6.1436 | 6.9171 | -0.7734 | 0.0406 |
| miR-362-3p | 4.7253 | 5.5853 | -0.8600 | 0.0290 |  | miR-193a-5p | 5.4007 | 5.8333 | -0.4325 | 0.0425 |
| miR-26b | 9.7553 | 10.2310 | -0.4756 | 0.0306 |  | miR-30a* | 6.8183 | 7.1728 | -0.3545 | 0.0496 |
| miR-361-3p | 6.1658 | 6.6966 | -0.5309 | 0.0319 |  |  |  |  |  |  |
| miR-192* | 4.3472 | 5.3080 | -0.9608 | 0.0373 |  |  |  |  |  |  |
| miR-660 | 6.3082 | 6.9920 | -0.6839 | 0.0414 |  |  |  |  |  |  |
| miR-374a | 5.0107 | 5.6917 | -0.6810 | 0.0419 |  |  |  |  |  |  |
| miR-125a-5p | 7.6032 | 8.2983 | -0.6951 | 0.0485 |  |  |  |  |  |  |
