# Supplementary material for: MicroRNA Profile Predicts Recurrence after Resection in Patients with Hepatocellular Carcinoma within the Milan Criteria
Source: PLoS One. 2011 Jan 27;6(1):e16435. doi: 10.1371/journal.pone.0016435 (PMC3029327; doi:10.1371/journal.pone.0016435)
Supplement: Table S7 — Differentially expressed microRNAs depending upon HCV status. * p-values of Student's T-test. Differentially expressed microRNAs with p<0.05 are listed. (DOC) [file pone.0016435.s010.doc]

Table S7

|  | T-miR | |  |  |  |  | N-miR | |  |  |
| --- | --- | --- | --- | --- | --- | --- | --- | --- | --- | --- |
| miR | HCV(+) | HCV(-) | Diff | p-value* |  | miR | HCV(+) | HCV(-) | Difference | p-value* |
| Up-regulated | |  |  |  |  | Up-regulated |  |  |  |  |
| miR-34b* | 6.4265 | 5.2253 | 1.2012 | 0.0003 |  | miR-222 | 6.8848 | 5.7984 | 1.0864 | <0.0001 |
| miR-30e* | 7.9329 | 7.2832 | 0.6497 | 0.0011 |  | miR-150 | 8.0739 | 6.9044 | 1.1695 | <0.0001 |
| miR-192* | 5.4894 | 4.3634 | 1.1260 | 0.0021 |  | let-7i | 9.2825 | 8.9220 | 0.3604 | 0.0008 |
| miR-455-3p | 8.6178 | 8.0202 | 0.5977 | 0.0036 |  | miR-181a | 8.0824 | 7.6304 | 0.4519 | 0.0008 |
| miR-193b | 9.1567 | 8.4048 | 0.7519 | 0.0092 |  | miR-886-3p | 8.0565 | 7.1551 | 0.9014 | 0.0016 |
| miR-215 | 9.5249 | 8.9718 | 0.5531 | 0.0167 |  | miR-96 | 3.8601 | 2.6540 | 1.2061 | 0.0030 |
| miR-34a | 9.7944 | 9.2538 | 0.5406 | 0.0187 |  | miR-106b | 9.1893 | 8.6924 | 0.4969 | 0.0032 |
| miR-30a | 10.6001 | 10.2103 | 0.3898 | 0.0188 |  | miR-155 | 5.7522 | 4.6028 | 1.1494 | 0.0033 |
| miR-365 | 8.3473 | 7.7817 | 0.5656 | 0.0196 |  | miR-342-3p | 8.8314 | 8.3948 | 0.4366 | 0.0033 |
| miR-125a-3p | 7.6574 | 7.2840 | 0.3735 | 0.0353 |  | miR-142-5p | 8.0579 | 7.2124 | 0.8455 | 0.0048 |
| miR-30c | 11.0656 | 10.8115 | 0.2541 | 0.0463 |  | miR-199a-5p | 10.7131 | 10.2907 | 0.4224 | 0.0060 |
| miR-26b | 10.2583 | 9.9081 | 0.3502 | 0.0494 |  | miR-221 | 7.9253 | 7.4180 | 0.5073 | 0.0073 |
|  |  |  |  |  |  | miR-199b-3p | 10.9496 | 10.5542 | 0.3954 | 0.0090 |
|  |  |  |  |  |  | miR-146b-5p | 10.1462 | 9.7907 | 0.3555 | 0.0093 |
|  |  |  |  |  |  | miR-142-3p | 5.8838 | 4.9084 | 0.9755 | 0.0102 |
|  |  |  |  |  |  | miR-362-3p | 5.0836 | 4.2171 | 0.8665 | 0.0110 |
|  |  |  |  |  |  | miR-199a-3p | 11.0069 | 10.6066 | 0.4003 | 0.0116 |
|  |  |  |  |  |  | miR-331-3p | 6.3914 | 5.8842 | 0.5072 | 0.0233 |
|  |  |  |  |  |  | let-7d | 12.1539 | 11.9643 | 0.1897 | 0.0267 |
|  |  |  |  |  |  | miR-16 | 11.2669 | 11.0684 | 0.1985 | 0.0288 |
|  |  |  |  |  |  | miR-186 | 5.8385 | 5.2965 | 0.5420 | 0.0334 |
|  |  |  |  |  |  | miR-147 | 5.5457 | 5.0445 | 0.5012 | 0.0406 |
|  |  |  |  |  |  | miR-140-3p | 8.0675 | 7.8483 | 0.2191 | 0.0414 |
|  |  |  |  |  |  | miR-185 | 7.5071 | 7.2301 | 0.2770 | 0.0432 |
|  |  |  |  |  |  | miR-146a | 7.6435 | 7.1835 | 0.4600 | 0.0459 |
| Down-regulated | |  |  |  |  | Down-regulated | |  |  |  |
| miR-146a | 6.1684 | 7.1622 | -0.9938 | 0.0127 |  | miR-296-5p | 8.1440 | 9.1344 | -0.9904 | <0.0001 |
| miR-24 | 11.4161 | 11.6670 | -0.2509 | 0.0161 |  | miR-1913 | 8.1286 | 8.7266 | -0.5980 | 0.0004 |
| miR-370 | 4.9433 | 5.7049 | -0.7615 | 0.0178 |  | miR-148a | 10.1907 | 10.7925 | -0.6019 | 0.0008 |
| miR-23a | 11.8544 | 12.1104 | -0.2560 | 0.0240 |  | miR-422a | 7.7153 | 8.2547 | -0.5394 | 0.0014 |
| miR-744 | 7.2950 | 7.8370 | -0.5420 | 0.0369 |  | miR-1228 | 5.8350 | 6.5709 | -0.7359 | 0.0022 |
| miR-1228* | 9.6644 | 10.1021 | -0.4377 | 0.0389 |  | miR-1238 | 5.4915 | 6.2016 | -0.7101 | 0.0034 |
| miR-494 | 9.6574 | 10.1067 | -0.4493 | 0.0461 |  | miR-1201 | 8.9610 | 9.4083 | -0.4473 | 0.0039 |
| miR-575 | 6.1797 | 6.6792 | -0.4995 | 0.0493 |  | miR-99a | 10.8449 | 11.1329 | -0.2880 | 0.0068 |
|  |  |  |  |  |  | miR-625* | 5.5356 | 6.3972 | -0.8615 | 0.0070 |
|  |  |  |  |  |  | miR-365 | 7.6609 | 8.0547 | -0.3939 | 0.0081 |
|  |  |  |  |  |  | miR-940 | 6.8369 | 7.3257 | -0.4888 | 0.0104 |
|  |  |  |  |  |  | miR-1249 | 4.9838 | 5.7895 | -0.8058 | 0.0207 |
|  |  |  |  |  |  | miR-378 | 9.0651 | 9.4886 | -0.4235 | 0.0232 |
|  |  |  |  |  |  | miR-92a | 8.4412 | 8.6436 | -0.2024 | 0.0420 |
