# Supplementary material for: MicroRNA Profile Predicts Recurrence after Resection in Patients with Hepatocellular Carcinoma within the Milan Criteria
Source: PLoS One. 2011 Jan 27;6(1):e16435. doi: 10.1371/journal.pone.0016435 (PMC3029327; doi:10.1371/journal.pone.0016435)
Supplement: Table S8 — Differentially expressed microRNAs depending upon cellular grade. *: p-values of one-way ANOVA test. Differentially expressed microRNAs with p<0.05 are listed. (DOC) [file pone.0016435.s011.doc]

Table S8

|  | T-miRs | | |  |  |  |  |
| --- | --- | --- | --- | --- | --- | --- | --- |
| miR name | grade1 | grade2 | grade3 | diff (1 vs 2) | diff (1 vs 3) | diff (2 vs 3) | p-value* |
| miR-191 | 10.1177 | 10.5007 | 10.1918 | -0.3831 | -0.0742 | 0.3089 | 0.0039 |
| miR-126* | 7.4668 | 6.5014 | 6.3017 | 0.9654 | 1.1651 | 0.1998 | 0.0074 |
| miR-1915 | 7.6699 | 7.3598 | 6.3638 | 0.3101 | 1.3061 | 0.9960 | 0.0202 |
| miR-378 | 8.7756 | 8.0561 | 8.4477 | 0.7194 | 0.3279 | -0.3915 | 0.0217 |
| miR-455-5p | 6.2626 | 6.0735 | 4.9452 | 0.1891 | 1.3175 | 1.1284 | 0.0232 |
| miR-126 | 12.0775 | 11.6789 | 11.7130 | 0.3985 | 0.3644 | -0.0341 | 0.0234 |
| miR-486-3p | 7.0058 | 6.4565 | 5.6443 | 0.5493 | 1.3615 | 0.8122 | 0.0285 |
| miR-1228 | 6.1406 | 5.9577 | 5.0541 | 0.1828 | 1.0864 | 0.9036 | 0.0290 |
| miR-20a | 9.3135 | 9.3725 | 8.0935 | -0.0590 | 1.2199 | 1.2790 | 0.0397 |
| miR-923 | 13.3713 | 13.9565 | 13.5210 | -0.5852 | -0.1497 | 0.4355 | 0.0419 |
| miR-106b | 9.5858 | 9.9831 | 9.9092 | -0.3974 | -0.3235 | 0.0739 | 0.0425 |
| miR-744 | 7.8564 | 7.3688 | 6.8402 | 0.4876 | 1.0162 | 0.5286 | 0.0456 |
