# Supplementary material for: MicroRNA Profile Predicts Recurrence after Resection in Patients with Hepatocellular Carcinoma within the Milan Criteria
Source: PLoS One. 2011 Jan 27;6(1):e16435. doi: 10.1371/journal.pone.0016435 (PMC3029327; doi:10.1371/journal.pone.0016435)
Supplement: Table S9 — Recurrence related microRNAs in HBV-positive cases. Univariate Cox proportional hazard model identified microRNAs associated with poor (red) and better (blue) recurrent outcome, respectively. Top-twenty significant microRNAs with p-value <0.05 are listed. MicroRNAs (displayed in red) which hazard ratio is greater than 1 were correlated with frequent recurrence, and are potential oncomiRs. In contrast, microRNAs (shown in blue) with hazard ratio less than 1 were associated with good recurrence-free survivals, and would be a tumor-suppressor miRs. (DOC) [file pone.0016435.s012.doc]

Table S9

| **HBV(+) cases (n=12)** | | | | | | | | |
| --- | --- | --- | --- | --- | --- | --- | --- | --- |
| **T-miRs** | | | |  | **N-miRs** | | | |
| **Rank** | **microRNA** | **hazard ratio** | **p-value** |  | **Rank** | **microRNA** | **hazard ratio** | **p-value** |
| **1** | **miR-1913** | **0.0992** | **0.0057** |  | **1** | **miR-638** | **0.1125** | **0.0073** |
| **2** | **miR-663** | **0.2277** | **0.0069** |  | **2** | **miR-1202** | **0.6105** | **0.0112** |
| **3** | **miR-1909** | **0.1647** | **0.0106** |  | **3** | **let-7i** | **18.629** | **0.0133** |
| **4** | **miR-575** | **0.2499** | **0.0140** |  | **4** | **miR-99b** | **8.4818** | **0.0140** |
| **5** | **miR-638** | **0.2146** | **0.0167** |  | **5** | **miR-107** | **15.909** | **0.0159** |
| **6** | **miR-18b** | **2.1562** | **0.0205** |  | **6** | **miR-146b-5p** | **7.3577** | **0.0184** |
| **7** | **miR-18a** | **2.2643** | **0.0215** |  | **7** | **miR-223** | **5.9927** | **0.0188** |
| **8** | **miR-1469** | **0.2768** | **0.0243** |  | **8** | **miR-663** | **0.3757** | **0.0189** |
| **9** | **miR-1908** | **0.2849** | **0.0251** |  | **9** | **miR-130b** | **2.0950** | **0.0202** |
| **10** | **miR-20a** | **3.1284** | **0.0255** |  | **10** | **miR-96** | **1.7335** | **0.0206** |
| **11** | **miR-20b** | **2.7301** | **0.0267** |  | **11** | **miR-23a** | **16.157** | **0.0211** |
| **12** | **miR-455-3p** | **2.5900** | **0.0277** |  | **12** | **miR-484** | **8.7674** | **0.0218** |
| **13** | **miR-296-5p** | **0.2116** | **0.0277** |  | **13** | **miR-146a** | **4.9436** | **0.0220** |
| **14** | **miR-362-3p** | **2.3579** | **0.0314** |  | **14** | **miR-1909** | **0.1095** | **0.0230** |
| **15** | **miR-17** | **3.4918** | **0.0343** |  | **15** | **miR-27a** | **14.401** | **0.0269** |
| **16** | **miR-106a** | **3.1753** | **0.0346** |  | **16** | **miR-149*** | **0.2075** | **0.0292** |
| **17** | **miR-335** | **2.6876** | **0.0362** |  | **17** | **miR-1260** | **0.0136** | **0.0301** |
| **18** | **miR-92a** | **3.0776** | **0.0372** |  | **18** | **miR-122*** | **0.3034** | **0.0311** |
| **19** | **miR-29a** | **0.2359** | **0.0435** |  | **19** | **miR-23b** | **10.679** | **0.0320** |
| **20** | **miR-940** | **0.2839** | **0.0449** |  | **20** | **miR-192** | **0.2663** | **0.0332** |
