# Supplementary material for: MicroRNA Profile Predicts Recurrence after Resection in Patients with Hepatocellular Carcinoma within the Milan Criteria
Source: PLoS One. 2011 Jan 27;6(1):e16435. doi: 10.1371/journal.pone.0016435 (PMC3029327; doi:10.1371/journal.pone.0016435)
Supplement: Table S10 — Recurrence related microRNAs in HCV-positive cases. Univariate Cox proportional hazard model identified microRNAs associated with poor (red) and better (blue) recurrent outcome, respectively. Top-twenty significant microRNAs with p-value <0.05 are listed. MicroRNAs (displayed in red) which hazard ratio is greater than 1 were correlated with frequent recurrence, and are potential oncomiRs. In contrast, microRNAs (shown in blue) with hazard ratio less than 1 were associated with good recurrence-free survivals, and would be a tumor-suppressor miRs. (DOC) [file pone.0016435.s013.doc]

Table S10

| **HCV(+) cases (n=51)** | | | | | | | | |
| --- | --- | --- | --- | --- | --- | --- | --- | --- |
| **T-miRs** | | | |  | **N-miRs** | | | |
| **Rank** | **microRNA** | **hazard ratio** | **p-value** |  | **Rank** | **microRNA** | **hazard ratio** | **p-value** |
| **1** | **miR-100** | **0.4810** | **0.0008** |  | **1** | **miR-27a** | **6.7460** | **0.0004** |
| **2** | **miR-99a** | **0.5821** | **0.0012** |  | **2** | **miR-486-5p** | **0.4468** | **0.0008** |
| **3** | **miR-125b** | **0.5773** | **0.0048** |  | **3** | **miR-24** | **13.869** | **0.0013** |
| **4** | **miR-92b*** | **1.6247** | **0.0131** |  | **4** | **miR-96** | **1.5296** | **0.0016** |
| **5** | **miR-30c** | **0.3833** | **0.0194** |  | **5** | **miR-21** | **1.6457** | **0.0026** |
| **6** | **miR-1268** | **1.6946** | **0.0200** |  | **6** | **miR-18a** | **2.2505** | **0.0038** |
| **7** | **miR-575** | **1.5276** | **0.0210** |  | **7** | **miR-142-3p** | **1.5871** | **0.0040** |
| **8** | **miR-1275** | **1.3010** | **0.0221** |  | **8** | **miR-23a** | **5.8634** | **0.0042** |
| **9** | **miR-30e** | **0.6382** | **0.0230** |  | **9** | **miR-148a** | **0.5434** | **0.0050** |
| **10** | **miR-130b** | **0.6909** | **0.0236** |  | **10** | **miR-1238** | **0.5584** | **0.0057** |
| **11** | **miR-1246** | **1.2356** | **0.0252** |  | **11** | **miR-191** | **9.1921** | **0.0105** |
| **12** | **miR-129-5p** | **0.7183** | **0.0261** |  | **12** | **miR-222** | **1.8491** | **0.0109** |
| **13** | **miR-148b** | **0.7898** | **0.0333** |  | **13** | **miR-296-5p** | **0.5316** | **0.0116** |
| **14** | **miR-22** | **0.5544** | **0.0347** |  | **14** | **miR-103** | **6.3409** | **0.0116** |
| **15** | **miR-103** | **0.6878** | **0.0375** |  | **15** | **let-7f** | **6.3824** | **0.0124** |
| **16** | **miR-99b** | **0.6843** | **0.0411** |  | **16** | **miR-18b** | **1.6347** | **0.0144** |
| **17** | **miR-638** | **1.4609** | **0.0455** |  | **17** | **miR-107** | **6.1065** | **0.0173** |
| **18** | **miR-665** | **1.2995** | **0.0485** |  | **18** | **miR-30c** | **0.4300** | **0.0179** |
| **19** |  |  |  |  | **19** | **miR-146b-5p** | **2.6044** | **0.0186** |
| **20** |  |  |  |  | **20** | **miR-378** | **0.6372** | **0.0187** |
