# Supplementary material for: MicroRNA Profile Predicts Recurrence after Resection in Patients with Hepatocellular Carcinoma within the Milan Criteria
Source: PLoS One. 2011 Jan 27;6(1):e16435. doi: 10.1371/journal.pone.0016435 (PMC3029327; doi:10.1371/journal.pone.0016435)
Supplement: Table S11 — Recurrence related microRNAs in hapatitis virus-negative cases. Univariate Cox proportional hazard model identified microRNAs associated with poor (red) and better (blue) recurrent outcome, respectively. Top-twenty significant microRNAs with p-value <0.05 are listed. MicroRNAs (displayed in red) which hazard ratio is greater than 1 were correlated with frequent recurrence, and are potential oncomiRs. In contrast, microRNAs (shown in blue) with hazard ratio less than 1 were associated with good recurrence-free survivals, and would be a tumor-suppressor miRs. (DOC) [file pone.0016435.s014.doc]

Table S11

| **HBV(-) HCV(-) cases (n=14)** | | | | | | | | |
| --- | --- | --- | --- | --- | --- | --- | --- | --- |
| **T-miRs** | | | |  | **N-miRs** | | | |
| **Rank** | **microRNA** | **hazard ratio** | **p-value** |  | **Rank** | **microRNA** | **hazard ratio** | **p-value** |
| **1** | **miR-17** | **30.384** | **0.0080** |  | **1** | **miR-30b** | **0.0170** | **0.0071** |
| **2** | **miR-106a** | **24.516** | **0.0109** |  | **2** | **miR-342-3p** | **0.0068** | **0.0189** |
| **3** | **miR-20a** | **29.072** | **0.0139** |  | **3** | **let-7e** | **32.803** | **0.0233** |
| **4** | **miR-93** | **48.859** | **0.0143** |  | **4** | **miR-147** | **147.4** | **0.0235** |
| **5** | **miR-92a** | **42.274** | **0.0148** |  | **5** | **miR-152** | **12.138** | **0.0249** |
| **6** | **miR-103** | **17.182** | **0.0154** |  | **6** | **miR-1202** | **0.5826** | **0.0352** |
| **7** | **miR-92b** | **16.617** | **0.0186** |  | **7** | **miR-126*** | **38.122** | **0.0384** |
| **8** | **miR-125b** | **0.0234** | **0.0191** |  | **8** | **miR-128** | **31.80** | **0.0389** |
| **9** | **miR-107** | **17.215** | **0.0193** |  | **9** | **miR-17** | **54.282** | **0.0416** |
| **10** | **miR-24** | **0.0780** | **0.0209** |  | **10** | **miR-93** | **25542** | **0.0468** |
| **11** | **miR-19a** | **8.6331** | **0.0227** |  | **11** | **miR-146a** | **0.0050** | **0.0481** |
| **12** | **miR-140-3p** | **0.0400** | **0.0230** |  | **12** | **miR-26b** | **58.694** | **0.0484** |
| **13** | **miR-20b** | **63.800** | **0.0260** |  | **13** | **miR-335** | **17.668** | **0.0492** |
| **14** | **miR-483-3p** | **1.4653** | **0.0272** |  | **14** | **miR-374b** | **8.9145** | **0.0497** |
| **15** | **miR-98** | **4.3393** | **0.0349** |  | **15** |  |  |  |
| **16** | **let-7f** | **1122.9** | **0.0357** |  | **16** |  |  |  |
| **17** | **miR-224** | **1.8818** | **0.0407** |  | **17** |  |  |  |
| **18** | **miR-148b** | **6.4607** | **0.0415** |  | **18** |  |  |  |
| **19** | **miR-30e** | **3.6760** | **0.0431** |  | **19** |  |  |  |
| **20** | **miR-125a-5p** | **0.2555** | **0.0456** |  | **20** |  |  |  |
