# Supplementary material for: MicroRNA Profile Predicts Recurrence after Resection in Patients with Hepatocellular Carcinoma within the Milan Criteria
Source: PLoS One. 2011 Jan 27;6(1):e16435. doi: 10.1371/journal.pone.0016435 (PMC3029327; doi:10.1371/journal.pone.0016435)
Supplement: Table S12 — Recurrence related microRNAs in grade 1–2 HCC cases. Univariate Cox proportional hazard model identified microRNAs associated with poor (red) and better (blue) recurrent outcome, respectively. Top-twenty significant microRNAs with p-value <0.05 are listed. MicroRNAs (displayed in red) which hazard ratio is greater than 1 were correlated with frequent recurrence, and are potential oncomiRs. In contrast, microRNAs (shown in blue) with hazard ratio less than 1 were associated with good recurrence-free survivals, and would be a tumor-suppressor miRs. (DOC) [file pone.0016435.s015.doc]

Table S12

| **Grade 1 (well diff, n=21)** | | | |  | **Grade 2 (mod diff, n=45)** | | | |
| --- | --- | --- | --- | --- | --- | --- | --- | --- |
| **Rank** | **T-miRs** | **hazard ratio** | **p-value** |  | **Rank** | **T-miRs** | **hazard ratio** | **p-value** |
| **1** | **miR-224** | **2.1100** | **0.0049** |  | **1** | **miR-99a** | **0.5806** | **0.0007** |
| **2** | **miR-193b** | **3.3817** | **0.0074** |  | **2** | **miR-100** | **0.5640** | **0.0015** |
| **3** | **miR-152** | **0.2691** | **0.0142** |  | **3** | **miR-378** | **0.6030** | **0.0092** |
| **4** | **miR-452** | **1.6740** | **0.0219** |  | **4** | **miR-30e*** | **0.5498** | **0.0186** |
| **5** | **miR-191** | **0.1882** | **0.0252** |  | **5** | **miR-129-5p** | **0.7036** | **0.0291** |
| **6** | **miR-20a** | **2.1191** | **0.0260** |  | **6** | **miR-140-3p** | **0.5061** | **0.0300** |
| **7** | **miR-92b** | **2.0843** | **0.0270** |  | **7** | **miR-422a** | **0.7489** | **0.0352** |
| **8** | **miR-1228** | **2.1790** | **0.0288** |  | **8** | **miR-99b** | **0.6989** | **0.0406** |
| **9** | **miR-199a-5p** | **0.6349** | **0.0298** |  | **9** | **miR-125b** | **0.8529** | **0.0468** |
| **10** | **miR-20b** | **2.0342** | **0.0325** |  | **10** | **miR-193b** | **0.7259** | **0.0493** |
| **11** | **miR-376c** | **0.7592** | **0.0372** |  |  |  |  |  |
| **12** | **miR-145** | **0.3157** | **0.0438** |  |  |  |  |  |
| **13** | **miR-199b-3p** | **0.6717** | **0.0454** |  |  |  |  |  |
